# Stereoselective synthesis of heterocyclic tetraphenylethylene analogues with configuration-dependent solid-state luminescence†

**DOI:** 10.1039/d4sc08333d

**Published:** 2025-05-05

**Authors:** Mathilde Seinfeld, Jean Rouillon, Raphael Rullan, Erwann Jeanneau, Stephan N. Steinmann, Chantal Andraud, Tangui Le Bahers, Cyrille Monnereau

**Affiliations:** a ENS de Lyon, CNRS, LCH, UMR 5182 69342 Lyon Cedex 07 France mathilde.seinfeld@ens-lyon.fr tangui.le_bahers@ens-lyon.fr; b Centre de Diffractométrie Henri Longchambon, Université Claude Bernard Lyon 1 5 Rue de la Doua 69100 Villeurbanne France; c Institut Universitaire de France 5 rue Descartes 75005 Paris France

## Abstract

While nowadays ubiquitous in a variety of optoelectronic applications, fluorophores displaying aggregation induced emission (AIE) and in particular those constructed around the tetraphenylethylene (TPE) core suffer severe limitations. In particular, it has been reported in many instances that stereoconfiguration around the central double bond may severely impact the solid-state luminescence properties (maximal emission wavelength and fluorescence quantum yield). Stereoselective synthesis of extended TPE cores remains challenging, and separation of diastereoisomer mixtures is generally tedious. In this paper, we introduce ditriazolostilbene moities (DTS) as an alternative to TPE. DTS offers two significant advantages over its TPE counterpart: firstly, a fully stereoselective synthesis of the (*E*)-isomer, and secondly, the use of a copper-catalyzed azide–alkyne cycloaddition (CuAAc) reaction in the final step, which simplifies access to novel derivatives. We illustrate the benefits of this approach using stereopure and (*E*) and (*Z*)-aggregates, powders and crystals of the molecule and show that emission properties are considerably dependent on their stereoconfiguration.

## Abstract 2

Stereopure AIEgens based on a ditriazolostilbene core are synthesized, enabling *E*/*Z* isomer comparison with detailed investigation of their solid-state luminescence and elucidation of the origin of their photophysical behavior.

## Graphical abstract

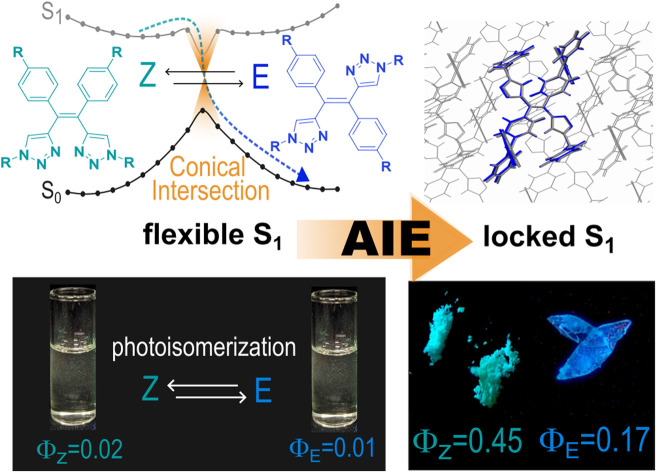

## Introduction

Although a wealth of luminescent molecules have been synthesized and studied by chemists for more than a century, their properties have been essentially investigated in solution. Solid state luminescence, which is quenched for most molecules, has been comparatively largely overlooked.^1^ However, the advent of optoelectronics in the last decades has changed this paradigm, as solid-state luminescent molecules and materials have become key components in a variety of display and imaging devices. Among those solid-state fluorophores, compounds for which fluorescence increases in the solid state are often referred to as Aggregation Induced Emission (AIE)^2^ emitters in the literature. AIE emitters have led to the development of new technologies, including nanoprobes, smart materials, and anticounterfeiting devices.^3–5^ Tetraphenylethylene (TPE) serves as a cornerstone for designing AIE-active molecules, as its remarkable solid-state properties are generally preserved upon functionalization.^3^

**Fig. 1:**
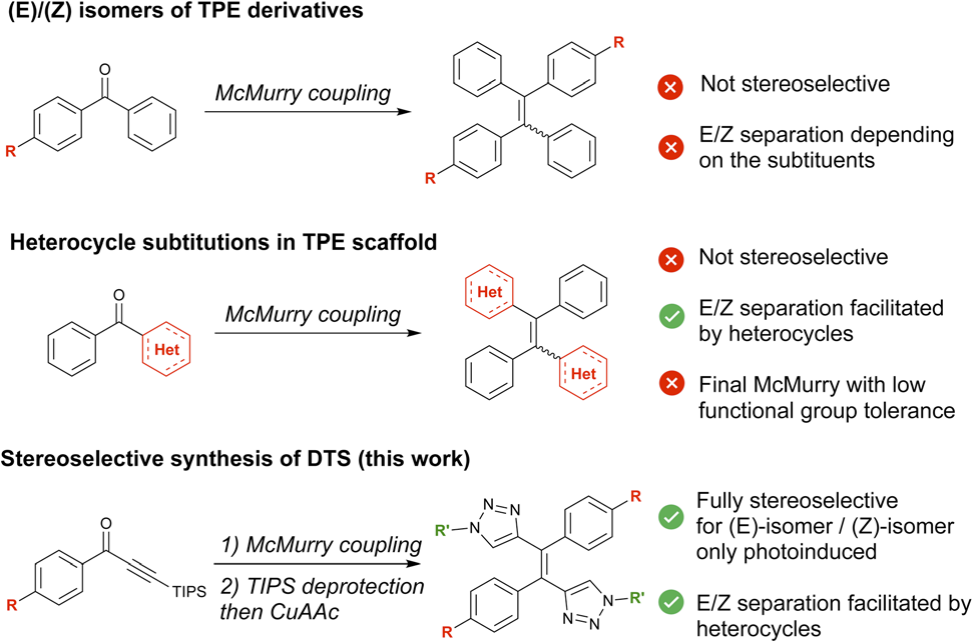
Different strategies to obtain (*E*/*Z*) isomers of TPE derivative.

When extended TPE derivatives bearing substituents on different phenyl sites are considered, stereoselectivity issues regarding the (*E*/*Z*) conformation of the ethylene bond may arise. This stereoconfiguration has a critical influence on the performances of the molecules.^4^ The 3D structure of each isomer will indeed determine the crystal packing,^5^ the aggregation morphology^6^ and thus strongly influence the luminescence features.^7^ Some sensing properties such as vapochromism,^8^ mechanoresponsive luminescence,^9,10^ and molecular recognition^11^ also rely on the crystal packing specificities, and are isomer dependent. These examples illustrate the diversity of targets and potential applications that can be attained simply by (*E*/*Z*)-isomerism. Unfortunately, the (*E*) and (*Z*) diastereoisomers of TPE derivatives are often thermodynamically very close in energy making stereoselective synthesis difficult to achieve.^12^ Most TPE derivatives are obtained through a key McMurry coupling step. This generally leads to mixtures of the (*E*) and (*Z*)-isomers^13^ (Fig. 1), although few strategies to specifically generate stereopure TPE derivatives have been described.^14–16^ Moreover, the separation of this diastereomeric mixture can prove extremely tricky using conventional chromatography techniques. One solution to overcome this difficulty has been to attach very polar^10^ or bulky groups^17,18^ to the phenyl groups around the double bond. This yields two diastereoisomers with different relative affinities for a given chromatographic separation phase. To create (*E*) and (*Z*)-isomers with sufficiently distinct properties to separate them, another less commonly used strategy involves the partial substitution of central phenyl groups by heteroaromatics^4^ (Fig. 1). Retaining the AIE of the TPE-like core, the introduction of heteroaromatics also gives rise to new properties such as photochromism^19–23^ or ion-sensing.^24,25^ Nevertheless, the significant synthetic modifications required for the introduction of heteroaromatics explain the limited number of examples of stereopure AIEgens achieved by this method.

In this work, aiming at developing a more universal methodology towards the synthesis of stereopure AIEgens, we designed a ditriazolostilbenes (DTS) core as an alternative to TPE by replacing two of the phenyl rings by triazole rings on either side of the central C=C double bond. DTS-1 and DTS-2 could be readily obtained in their pure (*E*)-configuration by means of a key stereoselective McMurry step and use of high-yielding copper-catalyzed azide–alkyne cycloaddition (CuAAc) click chemistry. Just like their TPE counterparts, we show that the newly synthesized compounds present AIE properties stemming from a dissipative photo-isomerization in solution, which is blocked in the solid state. In the case of some DTS molecules synthesized in this work, this photoisomerization process can be used for synthetic purposes: photoinduced (*E*/*Z*) mixture can indeed be easily separated *via* column chromatography providing access to the molecules in their pure (*Z*)-configuration, affording comparison with the primarily synthesized (*E*)-form. Spectroscopic solid state and AIE properties of these compounds are thus studied in detail, and rationalized on the basis of quantum chemical modelling and crystallographic characterization, revealing the potential interest of the scaffold towards solid state fluorescent emitters for bioimaging, in particular.

## Results and discussions

### Synthesis

**Fig. 2:**
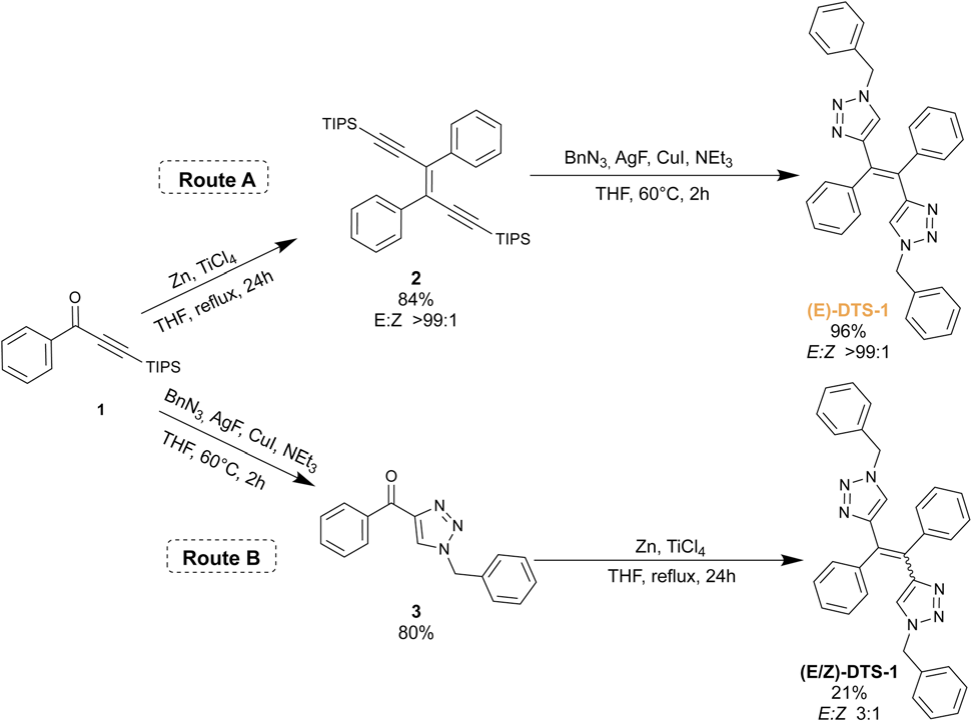
Synthesis of DTS-1 following two different synthetic paths.

The synthetic scheme for the ditriazolostilbene target is presented in Fig. 2. The route A displays the stereoselective method: McMurry reaction on a triisopropylsilyl (TIPS) protected phenylpropyn-1-one 1 led to the corresponding pure (*E*)-enediyne 2 when conducted in the dark (note: similar experiments performed with a TMS protecting group yielded the target compound in a 5 : 1 (*E*/*Z*) ratio, in agreement with initial report^26^). Upon *in situ* deprotection with silver(i) fluoride (AgF),^27^ the alkyne moieties reacted with benzyl azide under CuAAc conditions to yield the target compound (*E*)-DTS-1, with a retention of the initial (*E*)-configuration when protected from light. Noteworthily, statistical mixture of the (*Z*) and (*E*)-stereoisomers of DTS-1 was obtained when the CuAAc step was performed first, followed by McMurry coupling (Fig. 2, Route B). This demonstrates the crucial role of the TIPS group in McMurry coupling, which by its large steric hindrance favours exclusive formation of the (*E*)-isomer of DTS. The resulting (*Z*/*E*)-DTS-1 mixture (Fig. S5†) was characterized by the shift of the benzylic CH_2_ peak in proton NMR (from 5.30 ppm for the (*E*)-isomer to 5.42 ppm for the (*Z*)-isomer), allowing to characterize a 1 : 3 (*Z*/*E*) ratio. In this case, pure (*Z*)-isomer could not be isolated from the mixture using standard chromatographic techniques.

**Fig. 3:**
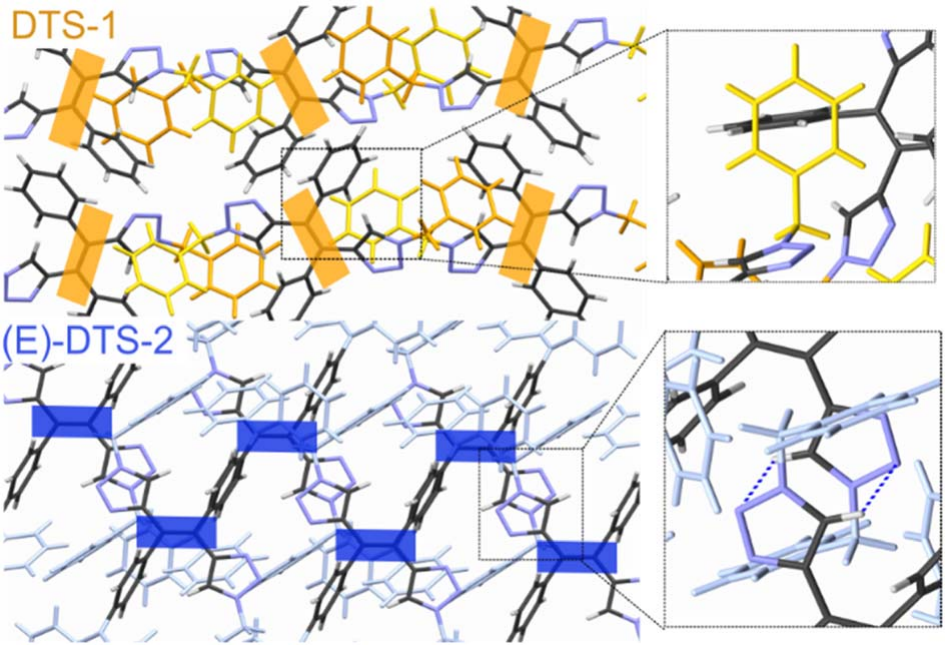
Crystal structure of (*E*)-DTS-1 (top) and (*E*)-DTS-2 (bottom). Central double bonds are highlighted with a rectangle to show specific packing: herringbone (orange, (*E*)-DTS-1) and β-motif (blue, (*E*)-DTS-2). Zooms show specific intermolecular interactions: T-contacts in DTS-1 and triazole π-stacking in (*E*)-DTS-2.

Stereoconfiguration was further verified by single crystal XRD structure determination (Fig. 3, top). Crystal structure shows a herringbone pattern relative to the arrangement of the central double bonds. Notable intermolecular contacts are T-shaped π-stacking (Fig. 3, zoom) between central phenyl and the benzene unit (in yellow), and intertwined triazole/benzyl units in neighbouring monomers.

**Fig. 4:**
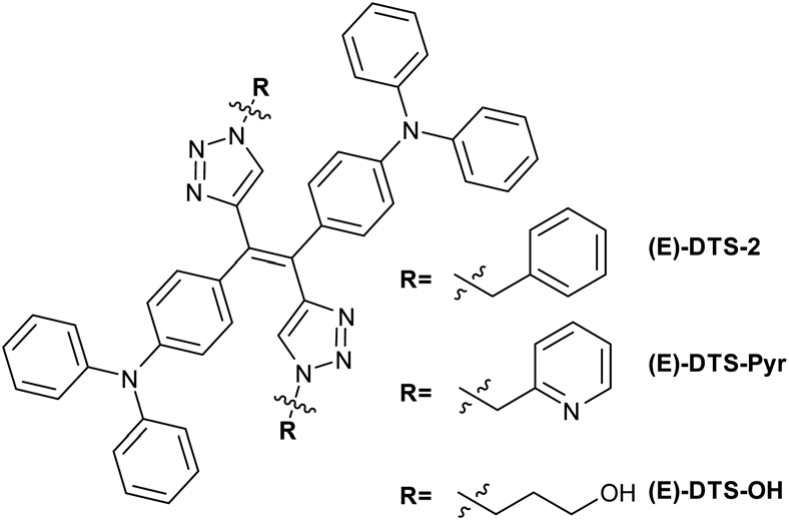
Structure of the synthesized diphenylamino-ditriazolostilbene derivatives.

**Fig. 5:**
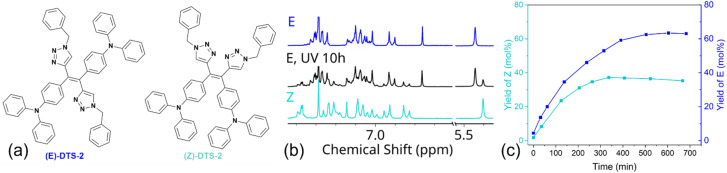
(a) Structure of the isolated diastereoisomers of DTS-2 (b) partial ^1^H NMR spectra of (*E*)-DTS-2 (blue), of irradiated (*E*)-DTS-2 (black) and (*Z*)-DTS-2 (cyan). (c) Isomerization yield of the initially pure (*E*) (blue) and (*Z*) (cyan) in CD_2_Cl_2_ (10^−3^ M) upon photoirradiation at 365 nm (4.7 mW cm^−2^) determined by NMR.

An extended version of the ditriazolostilbene chromophore featuring diphenylamino substituents on the *para*-positions of the phenyl ring DTS-2 (Fig. 4 and 5) was synthesized in its pure (*E*)-form, using a similar synthetic strategy. In that case, *in situ* AgF deprotection turned out ineffective. Therefore, TBAF deprotection of the TIPS groups was performed as a first step. The isolated enediyne intermediate was then submitted to CuAAc with benzyl azide. Again, this afforded pure (*E*)-DTS-2 when protected from light. The stereoconfiguration was again confirmed by crystal structure determination (Fig. 3, bottom). In this case, crystalline arrangement consists of stacked units in a β-motif,^28^ with π-stacking of triazole units between monomers.

Stereoselectivity of the CuAAC on the stereopure enedyine intermediate was further confirmed by the synthesis of a (*E*) benzyl pyridine and a (*E*) propanol ditriazolostilbene derivative (Fig. 4, respectively (*E*)-DTS-Pyr and (*E*)-DTS-OH). Determination of the stereoconfiguration was based on the characteristic chemical shift of the triazole proton, which was analogue in both case to that of (*E*)-DTS-2 (Fig. S15 and S17†).

Building on our previous work with TPE derivatives,^29^ we hypothesized that prolonged irradiation of a pure diastereoisomer of DTS-2 could induce partial photoisomerization, leading the system to reach a photostationary state. Therefore, synthesis of (*Z*)-DTS-2 was envisioned through exposure of a solution of (*E*)-DTS-2 to ambient light for several days leading to progressive isomerization of the mixture towards its photostationary state (*E*/*Z* 65 : 35) (Fig. 5b). Unlike DTS-1 and previously reported similar TPE–TPA derivative,^13^ the two isomers were easily separated by column chromatography owing to a higher polarity of the peripheral triazole substituent. The possibility to change these peripheral substituents offers an interesting degree of freedom to facilitate the (*E*/*Z*) separation. All ^1^H and ^13^C NMR spectra of the pure (*Z*) and (*E*)-forms of DTS-2 are featured as ESI (Fig. S1–S14).† Attempts at crystallizing (*Z*)-DTS-2 remained unsuccessful as this isomer tends to rapidly form small needles unsuitable for X-ray structure determination.

After having conducted this initial qualitative experiment to assess the feasibility of photoinduced (*E*/*Z*) isomerization in DTS derivatives, the kinetics of the (*E*/*Z*) photoisomerization process were monitored using proton NMR. The isomerization was performed starting from both pure (*Z*) and (*E*)-diastereoisomers of DTS-2, and photoisomerization yield was quantified by integrating the benzylic peak (chemical shift of 5.44 ppm in the (*E*)-form, shifted downfield by 0.04 ppm in the (*Z*)-form, as shown in Fig. 5) to draw quantitative conclusions. It was found that although photoisomerization was effective, it proceeded with a slow kinetic. This slow kinetic makes it more convenient for handling and maintaining stereopure compounds compared to TPE derivatives.^29^ As it could be expected, final isomer ratios are identical starting either from the (*E*) or (*Z*)-forms, confirming a photostationary equilibrium corresponding to a (*E*/*Z*) ratio of 65 : 35. The slight decline in the yield of (*Z*) monitored after reaching the photostationary state (between 300 and 700 min irradiation time, Fig. 5c) may be due to minor photodegradation, as evidenced by the appearance of NMR signals at longer times, indicating the formation of small amounts of other side photoproducts (not shown). An estimate of the photoisomerization quantum yield for all compounds was achieved using the initial regime approximation where the back isomerization is not considered.^30^

Photoisomerization quantum yield of (*E*)-DTS-2 to (*Z*)-DTS-2 was thus estimated as *Φ*_*E*→*Z*_ = 0.003 and was found similar for the reverse reaction *Φ*_*Z*→*E*_ = 0.004 (Fig. S20–S22†). Photoisomerization data for (*E*)-DTS-1 revealed a quantum yield (*Φ*_*E*→*Z*_) of 0.03, which, although lower than that of TPE analogues, is an order of magnitude higher than its triarylamine-extended derivative. This clearly indicates a non-innocent role of the electron-donating substituents in influencing the photophysical process, already observed on other olefinic systems.^31,32^

### Spectroscopic study

#### Spectroscopy in solution

**Fig. 6:**
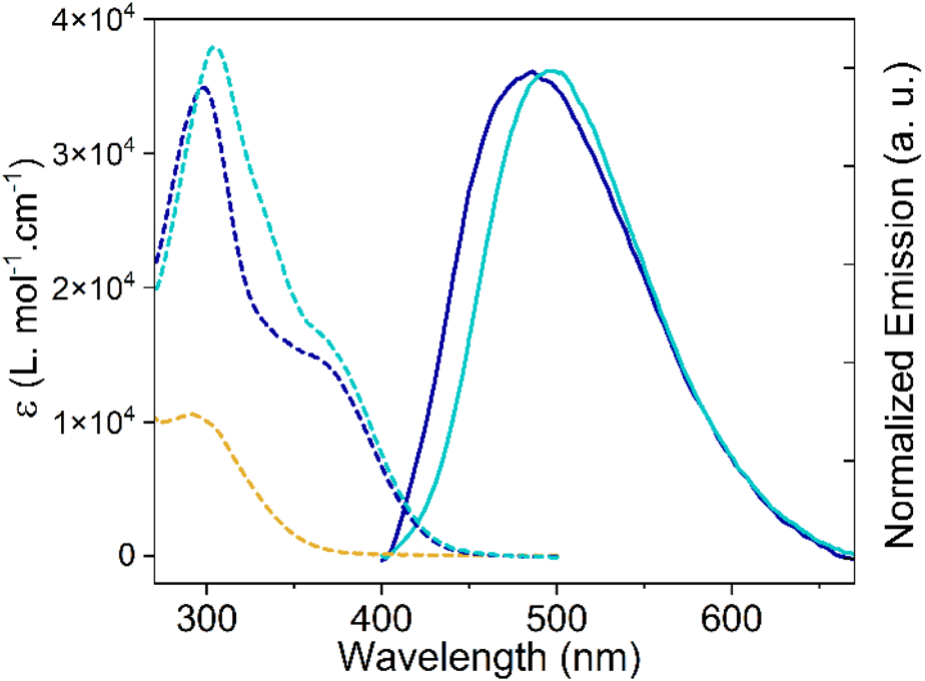
Absorption (dashed lines) and normalized emission (solid lines) for (*E*)-DTS-1 (yellow curve), (*E*)-DTS-2 (blue curve), (*Z*)-DTS-2 (cyan curve) in THF, 10^−5^ M. Note that no fluorescence is observed for (*E*)-DTS-1.

Spectroscopic studies of (*E*)-DTS-1 and the (*E*) and (*Z*) isomers of DTS-2 were first conducted in 10^−5^ M THF solutions (Fig. 6 and Table 1), while corresponding TD-DFT calculations and natural transition orbital (NTO) analysis are reported on Tables S3–S5.† In the case of (*E*)-DTS-1, a single structureless absorption band is seen at *ca.* 295 nm due to a π–π* transition delocalized on the whole π-system. This π–π* transition is redshifted in (*E*)-DTS-2 and (*Z*)-DTS-2 due to a donating contribution from the introduced triphenylamine groups, resulting in an absorption band at 375 nm. The most intense band in (*E*)-DTS-2 and (*Z*)-DTS-2 spectra, at 300 nm, comes from two local π–π* transitions mostly centered on each triphenylamine group. This absorption band is slightly blue shifted in (*E*)-DTS-2 due to an additional π–π* transitions close in energy which is not found for (*Z*)-DTS-2 (Fig. S31†). Another difference between the (*E*) and (*Z*) isomer absorption spectra is the extra shoulder on the (*Z*)-DTS-2 absorption spectrum around 334 nm. TD-DFT shows that this shoulder corresponds to a π–π* transition from the triphenylamine groups to the ethylenic double bond which is absent in (*E*)-DTS-2.

**Table 1:** Experimental absorption and emission *λ*_max_ wavelengths with the corresponding fluorescence quantum yield (*Φ*_f_) in solution (THF), in nanoprecipitates (90/10 water/THF) and in powder

Compound	State	Absorption *λ*_max_ (nm)	Emission *λ*_max_ (nm)	*Φ*_f_
(*E*)-DTS-1	Solution	295	—	—a
	Nanoprecipitate	288	—	—a
	Powder	—	500	0.05
	Ground powder	—	479	0.05
(*E*)-DTS-2	Solution	300, 375	487	0.008
	Nanoprecipitate	305, 346	526	0.33
	Powder α	—	550	0.09
	Powder β	—	450	0.17
	Ground powder β	—	482	0.05
(*Z*)-DTS-2	Solution	302, 334, 375	497	0.016
	Nanoprecipitate	308, 385	535	0.39
	Powder	—	500	0.45

a No detectable emission.

As generally observed with most reported TPE analogues, (*E*)-DTS-1 is found not to be emissive in solution while DTS-2 isomers exhibit only faint fluorescence in THF (*Φ*_f__(_*_E_*_)-DTS-2_ = 0.008; *Φ*_f__(_*_Z_*_)-DTS-2_ = 0.016). In the latter case, the emission profile was very similar for both isomers with only a slight red shift in the emission band (*λ*_*E*_ = 487 nm, *λ*_*z*_ = 497 nm) which we attribute to the dipolar nature of the (*Z*)-isomer. (*Z*)-DTS-2 indeed shows positive solvatochromism with a linear red-shift of the maxima of emission with the increased orientation polarizability of the solvent as well as positive solvatokinetics with a decrease of emission intensity in more polar solvents.^33^(*E*)-DTS-2 also shows positive solvatochromism of emission but its solvatokinetics is more erratic, associated with its quadripolar nature (Fig. S25†).

#### Nanoprecipitation

**Fig. 7:**
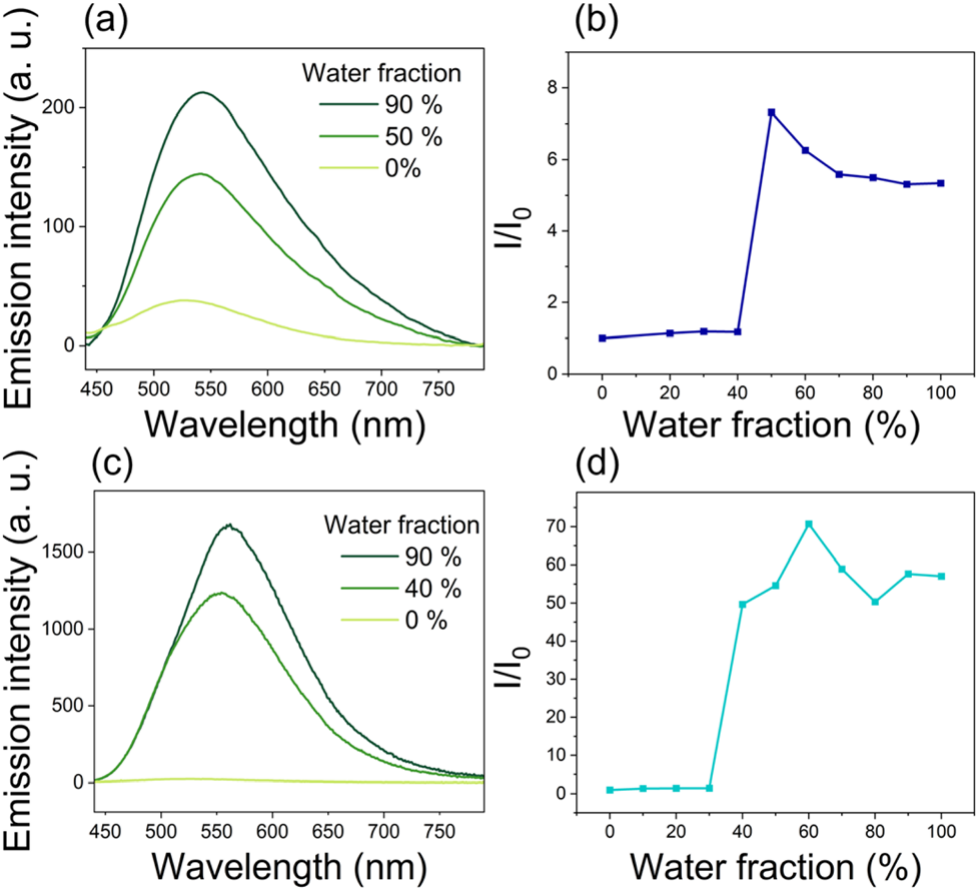
Emission spectra of (*E*)-DTS-2 (a) and (*Z*)-DTS-2 (c) with different DMSO/water fractions (*f*_w_) at 10^−5^ M. Plots of *I*/*I*_0_ of (*E*)-DTS-2 (b) and (*Z*)-DTS-2 (d), where *I* is the intensity at a given *f*_w_ and *I*_0_ is the initial intensity in DMSO.

To assess the AIE potential of our compounds, a nanoprecipitation method based on different DMSO/water mixtures was used (Fig. 7). (*E*)-DTS-1 remained non-emissive upon the addition of water, discarding it for further use as an AIE active compound. On the contrary, (*E*)-DTS-2 showed AIE behaviour with a 7-fold enhanced emission when the water fraction (*f*_w_) is above 40%. Comparatively, the AIE performances of the (*Z*)-DTS-2 isomer were consistently improved, since the luminescence was enhanced by a factor of 70 for *f*_w_ exceeding 30%. This larger AIE enhancement of the (*Z*) compared to the (*E*) isomers underline the key effect of the stereoconfiguration on the spectroscopic properties. This difference in AIE behaviour suggests major differences in the microscopic ordering of both compounds in their respective nanoprecipitate states. In addition to the enhanced luminescence, the aggregation causes a significant red shift of approximately 30 nm in the emission maxima for both isomers.

Similarly, (*E*)-DTS-Pyr and (*E*)-DTS-OH showed a sharp increase of luminescence upon addition of water (Fig. S27†), with respectively a 30-fold and 2.5-fold increase. We postulate that the presence of alcohol functions at the extremity of (*E*)-DTS-OH may enhance its solubility in water and therefore hinder the nanoprecipitation process. Overall, however, this confirms that the potential of this novel class of the diphenylamino-ditriazolostilbene structure for solid state emission and AIE related applications is retained upon functionalisation.

#### Solid-state emission

**Fig. 8:**
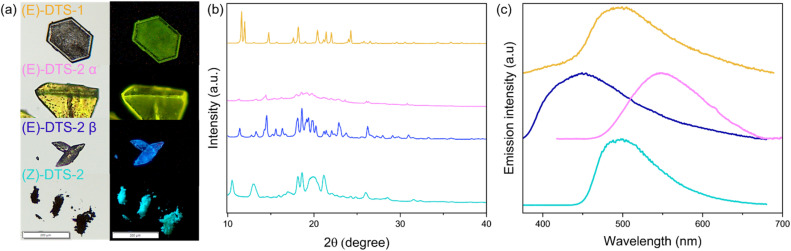
(a) Photoluminescence spectra of crystalline powder of (*E*)-DTS-1 (yellow), (*E*)-DTS-2 in α powder (pink), β powder (blue) and (*Z*)-DTS-2 (cyan). (b) XRD powder diffraction patterns for (*E*)-DTS-1 (yellow), (*E*)-DTS-2 in α (blue) and β (pink) and (*Z*)-DTS-2 (cyan). (c) Optical microscope images of (*E*)-DTS-1 (yellow), (*E*)-DTS-2 in α (blue) and β (pink) powder and (*Z*)-DTS-2 (cyan) under white light (left) and UV-light (right).

Next, emission properties of the compounds in their solid-state form were investigated (Table 1 and Fig. 8). Crystalline powders of each compound were obtained by evaporation of concentrated CH_2_Cl_2_/EtOAc mixture under vacuum. Contrary to the non-emissive nanoprecipitate, (*E*)-DTS-1 in crystalline powder is reasonably emissive (*Φ*_f__(_*_E_*_)DTS-1_ = 0.05 at *λ*_max_ = 500 nm). Powder XRD shows extremely narrow and well resolved peaks indicating high cristallinity of the powder sample (Fig. 7b). In line with this high degree of crystallinity, the spectrum almost perfectly matches the computed data obtained from single crystal XRD (Fig. S28†). This could in retrospect explain why no emission is observed in the nanoprecipitate, where the fast precipitation kinetic is prone to affect crystal packing. Thus, a highly ordered structured seems to be necessary to observe fluorescence for this compound.

In the case of (*E*)-DTS-2, crystalline powders of different macroscopic aspect were obtained, showing distinctive luminescence features when placed under UV lamp (Fig. 8a), which enabled us to investigate the influence of crystallinity on the luminescence. A yellow powder labelled α showed broad and small intensity peaks in powder XRD, indicating an amorphous character. The luminescence of (*E*)-DTS-2 powders can be compared with nanoprecipitates, where the crystallinity is unknown. While all samples exhibit similar emission intensities, there is a significant shift in emission wavelength: the crystalline powder, which XRD spectrum presents a relatively good agreement with the computed data modeled from single crystal XRD (Fig. S28†), shows a 50 nm blue shift in emission compared to the more amorphous powder and the nanoprecipitates. This indicates that changes in crystallinity strongly influence emission maxima. Notably, the emission maxima of the nanoprecipitates align with those of the amorphous powder, suggesting less organized packing in the nanoprecipitates, consistent with their kinetically driven formation. These large crystallinity-dependent variations of fluorescence signatures prompted us to investigate possible mechanochromic effects on the solid luminescence. Crystalline powder of (*E*)-DTS-1 and (*E*)-DTS-2 were finely ground, and their luminescence was compared to the pristine crystalline powders: in both cases, the ground structures showed a distinctively shifted luminescence, along with a moderate decrease in luminescence quantum yield (Table 1). Interestingly, in the case of (*E*)-DTS-2, luminescence of the ground powder showed a strong qualitative resemblance to that of isolated molecules in diluted solutions.

For (*Z*)-DTS-2, both microscopic observation of the powder and XRD diffraction peaks showed a less crystalline organization. In terms of luminescence, the solid-state quantum yield is higher than that of the (*E*)-DTS-2, similarly to what has been observed in their nanoprecipitate state. To our surprise, this difference of emission intensity from the two DTS-2 isomers does not seem to result from a univocal difference in degree of crystallinity, but rather be related to their respective stereoconfigurations. Indeed, (*Z*)-DTS-2 is significantly more emissive than both α and β forms of (*E*)-DTS-2 samples.

### Interpretation of the AIE origin

Although there is still no absolute consensus regarding the mechanism driving the AIE phenomenon, the paradigm has largely evolved in the specific case of TPE since the early models proposed by Tang and collaborators.^2^ Initial explanations for the vanishing emission efficiency in solution relied on dominant internal conversion and relaxation processes through rotational and vibrational motion of the peripheral phenyl groups that would be restricted in aggregates (Restriction of Intramolecular Motions, RIM).^8^ These explanations were not in line with pre-existing spectroscopic studies of the TPE molecule. Those early studies and following work characterized a π-twist of the double bond at the excited state^34,35^ and identified photoproducts from photocyclization and photoisomerization pathways.^29,36,37^ Computational evidence revealed the importance of conical intersections (CI)^38–40^ which lead to fast deexcitation and potentially the formation of photoproducts (Restriction of Access to Conical Intersection, RACI). The relative importance of photocyclization^41,42^ against the (*E*/*Z*) photoisomerization^43,44^ in the deexcitation process is still debated, especially given that most studied systems are symmetrical TPE where the (*E*/*Z*) photoisomerization cannot be monitored. As (*E*/*Z*) photoisomerization was clearly characterized by NMR monitoring of our compounds (Fig. 5), it can be expected that the weak luminescence observed in solution for the ditriazolostilbene derivatives of this study is due to competing photoisomerization processes.

To test this hypothesis, additional luminescence studies were performed. In a first set of experiments, the emission intensity of isoconcentrated solutions of (*E*)-DTS-1 and (*E*)-DTS-2 were recorded in THF, EtOH and glycerol (Fig. S29a and S30a†). In both cases, a marked (*ca.* 40–70 fold) luminescence enhancement was achieved in the higher viscosity solvent, witnessing a restriction of excited state rearrangements and ultimately access to conical intersection. Additional comparison of luminescence intensities in 2-methyltetrahydrofuran at room temperature and 77 K highlighted a similar effect: in the glass obtained at 77 K, luminescence of both compounds was markedly increased, again in line with our hypothesis of a restricted access to conical intersection (Fig. S29b and S30b†).

**Fig. 9:**
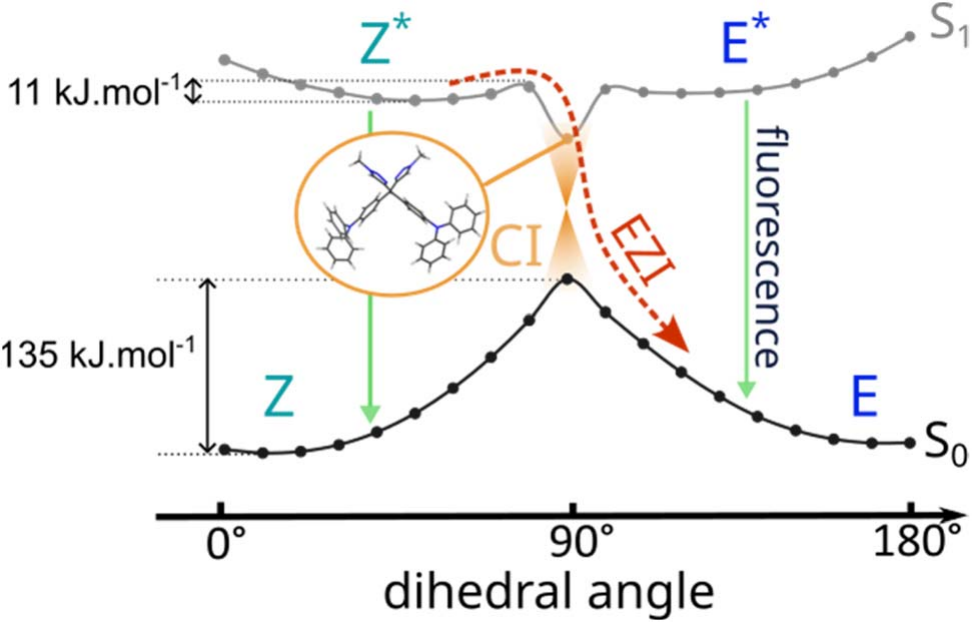
PES scans along the central double bond dihedral angle. At the ground state (black), a large energy barrier prevents thermal isomerization. At the first excited state (grey), two minima allowing for deexcitation *via* fluorescence (green pathway) coexist with a conical intersection (CI, orange) which results in (*E*/*Z*) photoisomerization (EZI, red pathway).

To further support this hypothesis, the potential energy surface (PES) of (*E*/*Z*)-DTS-2 isomers was explored using DFT and TD-DFT (Fig. 9). Relaxed scans along the dihedral angle of the ethylene bond, *i.e.* the bond that determines the (*E*) or (*Z*)-stereodescriptor, provide an estimate of the energy barriers for the isomerization process. At the ground state, two conformations for the double bond correspond to minima of the PES, one at 10°, the stable (*Z*)-isomer and one with at 170°, associated with the (*E*)-isomer. Both minima are separated by a large energy barrier of 135 kJ mol^−1^, which prevents any thermally induced isomerization at room temperature. The differences of energy between the two isomers, 11 kJ mol^−1^, is too small to conclude on a thermodynamically favoured isomer for this system on this single basis. This is in line with the isomerization ratio of 65/35 (*E*/*Z*) found experimentally, which according to Boltzmann distribution law, should be associated to an energy difference of approximately 3 kJ mol^−1^, within the error of DFT.

To understand the effect of light exposure on the system, PES exploration was further carried out at the first excited state S_1_. There, given the antibonding nature of the ethylenic double bond of the singly populated LUMO orbital, the minima geometries are more distorted. Again, in spite of the very shallow aspect of the PES, two local minima are obtained, at 50° and 120°. At those angles values, the geometries maintain (*E*) or (*Z*)-character even in the excited state, from which luminescence can occur. This result is in agreement with the distinct (*E*) and (*Z*)-isomer fluorescences observed in solution. Going towards 90°, at the *EZ* intersection, an energy barrier of around 11 kJ mol^−1^ is computed. Although small, this energy barrier defines a stability zone for the (*E*) and (*Z*)-isomers in the excited state. However, at 90°, another minimum of S_1_ is identified for a cross-shape geometry below the two emitting minima in terms of energy. This suggests the existence of a conical intersection near this geometry, which, as already established with TPE derivatives,^45^ provides an efficient non-radiative deexcitation pathway to the ground state, with possible outcome to either an (*E*) or a (*Z*)-configuration. This fast deexcitation *via* photoisomerization would compete with fluorescence, a slower radiative process, explaining the weak fluorescence of DTS-2 in solution. Similar results were found for the PES of DTS-1 (Fig. S33†). Experimental results suggest that deexcitation through the conical intersection is more efficient in DTS-1 as an absolutely dark excited state has been monitored for this molecule in solution, and a larger photoisomerization quantum yield was determined. However, on a computational viewpoint, calculation of the probability of deexcitation *via* one or the other pathways (fluorescence or CI) would require dynamical modelling such as non-adiabatic dynamic simulations,^46,47^ which is out of reach for large systems such as our molecules. This PES study in solution sheds light on the importance of the possibility of torsion of the central double-bond at the excited-state to explain the photoisomerization and associated quenched fluorescence in solution.

To finalize this analysis of PES at the excited state, two other chemical groups rotations involving the triphenylamine moieties were performed for DTS-2 showing no clue for a conical intersection. This supports the idea that rotation around the central ethylenic central double bond is the dominant process accounting for efficient non-emissive relaxations (Fig. S32–S34†).

**Fig. 10:**
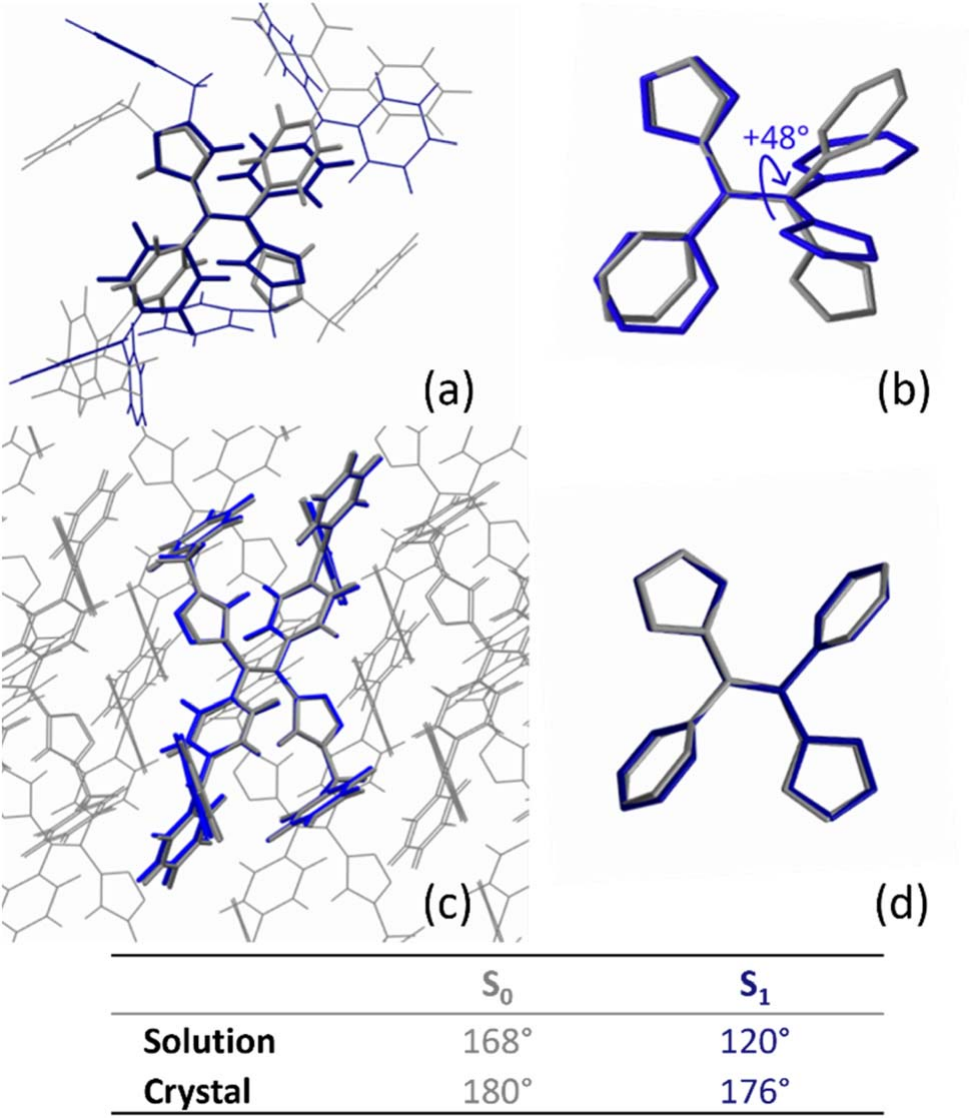
(top) Superposition of optimized structures of (*E*)-DTS-2 at the ground state (grey) and the first excited state (blue): (a) optimized in solution, (b) central phenyl and azide groups with hydrogen atoms omitted of the same structures, (c) optimized in crystal (d) central phenyl and azide groups with hydrogen atoms omitted of the same structures. (bottom) Table of computed ethylene dihedral angle values (°) of (*E*)-DTS-2 found at the minima of energy of the PES in solution and in crystal.

In the solid state, the possibility of torsion around that double bond was also probed thanks to the crystal structures obtained for (*E*)-DTS-1 and (*E*)-DTS-2. Starting from those crystallographic data, optimized structures in the condensed phase were computed at the ground and first excited state using periodic DFT and TD-DFT along with the CP2K quantum chemical code.^48–50^ Superposition of the most stable conformation at the ground and first excited states highlights the conformational relaxation that can take place upon excitation of the molecule in a given environment (Fig. 10a and c). Again, the dihedral angle of the central double bond provides a clear and simple parameter to assess this relaxation (Fig. 10b and d). Analogous analysis on (*E*)-DTS-1 yields similar results (Table S6†). For (*E*)-DTS-2, unlike in solution, the dihedral angle at the ground state in solid is close to 180°, imposing a flat conformation to the double bond. In the excited state, the difference between geometric reorganization taking place in solution and that enabled in the crystal becomes much more pronounced. While in solution the relaxed S_1_ geometry reaches a dihedral angle of 120° for (*E*)-DTS-2, in the crystal, this dihedral angle is locked at 176°. Steric hindrance within the crystal enforces a planar conformation of the double bond, mirroring that of the ground state. This highly constrained geometry could explain the blue shift of 37 nm observed for the emission of (*E*)-DTS-2 in the crystal as compared to that seen in solution, where relaxation processes to a local minimum of the PES are allowed. This locked dihedral also impedes the access to the conical intersection, located near 90°, which explains the strong amplification of emission efficiency at the solid state.

## Conclusions

In this work, we have successfully synthesized a pure (*E*) heterocyclic analogue of TPE *via* a combination of a key stereoselective McMurry reaction and high yielding CuAAc click chemistry. Through photoisomerization to the thermodynamic equilibrium and purification of the resulting stereoisomers mixture by column chromatography, the (*Z*)-form of this ditriazolostilbene was also isolated, enabling systematic comparison of the photophysics of both stereoisomers. In particular, the luminescence properties in aggregates, crystals and solution were compared for each isomer. Interestingly, the (*Z*)-isomer is consistently more luminescent than its (*E*)-counterpart. A shift in emission wavelength for the (*E*)-isomer in different solid phases was correlated to its degree of crystallinity. The PES scan performed at the TD-DFT level allowed to rationalize these results. The existence of two minima at the S_1_ state explains the weak residual emission recorded in solution, while the low energy barrier to reach the conical intersection provides an explanation for the low fluorescence efficiency and photoisomerization processes. In the crystal, geometry relaxation of the first excited confirmed that the rigidity imposed by the condensed phase constrains the geometrical distortion at the excited state leading to a blue shift in the emission and an enhance fluorescence efficiency.

This study of a new heterocyclic AIEgen can set a first step toward more application-centred molecular design. Retaining the AIE ability, the potential of functionalization offered by the CuAAc click reaction can be used to synthesize objects of higher complexity and control the molecular hindrance in solid state packing, a domain that we are currently exploring. In particular, we are currently investigating strategies that involve the quaternization of the triazole ring to enhance the red-emitting properties of resulting fluorescent organic nanoparticles, for application in two-photon fluorescent bioimaging. By fine-tuning the electronic structure through quaternization, we seek to optimize two-photon absorption cross-sections and fluorescence efficiency, ultimately enabling deeper tissue penetration and higher-resolution imaging, while improving biocompatibility. These studies are undergoing, and will be reported shortly.

## Data availability

The authors declare that the data supporting the findings of this study are available within the article and the ESI.†

## Author contributions

M. S.: conceptualization, formal analysis, investigation, visualization, writing – original draft, review and editing. J. R.: conceptualization, investigation, visualization, writing – review and editing. R. R.: investigation, writing – review and editing. E. J.: investigation, formal analysis – review and editing. S. N. S.: supervision. C. A.: project administration, writing – review and editing. T. L. B.: conceptualization, formal analysis, project administration, supervision, writing – review and editing. C. M.: conceptualization, project administration, supervision, writing – review and editing.

## Conflicts of interest

There are no conflicts to declare.

## Supplementary Material

SC-OLF-D4SC08333D-s001

SC-OLF-D4SC08333D-s002
