# Supplementary material for: Stereoselective synthesis of heterocyclic tetraphenylethylene analogues with configuration-dependent solid-state luminescence
Source: Chem Sci. 2025 May 5;16(23):10523–31. doi: 10.1039/d4sc08333d (PMC12070306; doi:10.1039/d4sc08333d)
Supplement: SC-016-D4SC08333D-s001 [file SC-016-D4SC08333D-s001.pdf]

Supplementary Information (SI) for Chemical Science.
This journal is © The Royal Society of Chemistry 2025

Stereoselective synthesis of heterocyclic tetraphenylethylene analogues with configuration-dependent solid-state luminescence

Mathilde Seinfeld,*[a] Jean Rouillon,[a] Raphael Rullan,[a] Erwann Jeanneau,[b] Stephan N. Steinmann,[a] Chantal Andraud,[a] Tangui Le Bahers*[a,c] and Cyrille Monnereau[a]

a. ENS de Lyon, CNRS, LCH, UMR 5182, 69342, Lyon cedex 07, France
b. Centre de Diffractométrie Henri Longchambon, Université Claude Bernard Lyon 1, 5 Rue de la Doua, 69100 Villeurbanne, France
c. Institut Universitaire de France, 5 rue Descartes, 75005 Paris, France

Table of Content

Synthesis 3

Compound characterization 10

Single crystal X-ray diffraction 19

Photophysical properties 22

  Photoisomerization quantum yield measurements 23

  Luminescence quantum yield measurements 24

  Solvatochromism 26

Computational methods 29

  Natural transition orbitals 30

  Computed absorption spectra. 32

  Dihedral scans 33

  Cartesian coordinates of computed structures 34

References 43

Synthesis

Starting materials were purchased from Sigma Aldrich®, Abcr® or TCI®. Synthesis were performed in an oven-dried vessel under argon atmosphere when relevant. Dry solvents were obtained from Solvent Purification System MB SPS5 from Mbraun. The reactions were monitored by analytical thin-layer chromatography (TLC) on a Merck 60 F-254 precoated silica gel plate (0.2 mm thickness), and the product was revealed by a UV lamp. Purification by column chromatography was made using over Merck silica gel 60 (0.040–0.063 mm) or aluminium oxide Brockmann I, neutral.

NMR spectra are recorded at room temperature on a Bruker Avance 300 or 400. Chemical shifts are reported as values (ppm) with reference to the peak of solvent. Abbreviation for the ^1H NMR data are as follows: chemical shift δ, multiplicity (s = singlet, d = doublet, t = triplet, q = quartet, m = multiplet), coupling constants J. High resolution mass spectrometry were performed at Centre Commun de Spectrométrie de Masse (Villeurbanne, France). Elemental analyses were performed at the Centre Régional de mesures physiques de l'Ouest (Rennes, France).

1-Phenyl-3-(triisopropylsilyl)-2-propyn-1-one

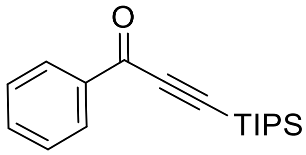

In a dry vessel, benzoyl chloride (0.4 mL, 3.56 mmol) was added to a solution of $Pd(PPh_3)_2Cl_2$ (0.043 g, 0.06 mmol), copper(I) iodide (0.031 g, 0.15 mmol) and trimethylamine (2.0 mL, 14.9 mmol) in THF (5 mL) at room temperature. Then (triisopropylsilyl)acetylene (0.7 mL, 2.97 mmol) was added to the mixture and stirred for 30 minutes. After treatment with saturated aqueous ammonium chloride (50 mL) and two extractions with diethyl ether (100 mL), the combined organic layers, solvents were removed under vacuum. Crude product was purified by SiO_2 column chromatography (PE: CH_2Cl_2, 9:1 to 3:2 (v/v)) to give compound (0.831 g, 98 %) as a colourless oil. ^1H NMR (300 MHz, CDCl3) δ 8.18 (d, J = 7.0 Hz, 2H), 7.65 – 7.58 (t, 1H, 7,4 Hz), 7.49(t, J = 7,3 Hz, 2H), 1.16 (m, 21H).

(*E*)-[(triisopropylsilyl)ethynyl]stilbene

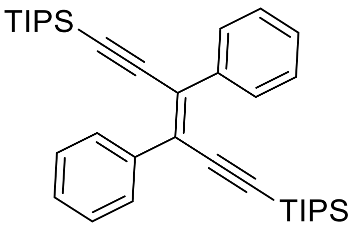

In a dry vessel, titanium tetrachloride (1M in CH_2Cl_2 8.4 mL, 8.73 mmol, 3 eq.) was added to solution of zinc (1g, 16.7 mmol 6 eq.) in dry THF (100 mL) at 0°C, then the mixture was refluxed for one hour. A solution of 1-Phenyl-3-(triisopropylsilyl)-2-propyn-1-one (0.8 g, 2.79 mmol, 1 eq.) in 20 mL of THF was added to the reaction mixture and refluxed overnight, away from light. The reaction was cooled with an ice-water bath and quenched by addition of saturated solution of K_2CO_3 (100 mL). The obtained slurry was filtered through plug of silica and rinse three times with CH_2Cl_2 (100 mL). Organic layer was

washed with brine and water, dried over Na_2SO_4, filtered and solvents were removed under vacuum. Crude product was purified by SiO_2 column chromatography (PE only to PE: CH_2Cl_2, 9:1 (v/v)) to give the desired olefin (0.634 g, 84%) obtained as a white powder. ^1H NMR (300 MHz, CDCl3) δ 8.02 – 7.82 (m, 4H), 7.50 – 7.01 (m, 6H), 1.00 (m, 42H). ^{13}C NMR (75 MHz, CDCl3) δ 139.24, 129.63, 129.39, 128.16, 127.82, 107.30, 102.45, 18.72, 11.49.

DTS-1

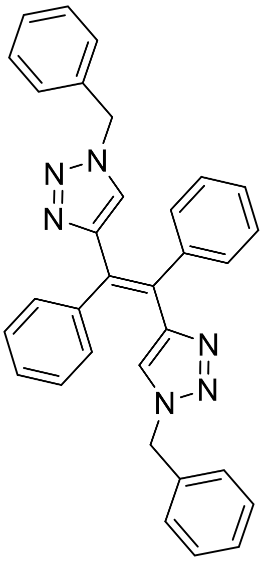

In a dry vessel, (*E*)-[(triisopropylsilyl)ethynyl]stilbene (0.1 g, 0.14 mmol), copper(I) iodide (0.047 g, 0.14 mmol) and silver(I) fluoride (0.1 g, 0.78 mmol) were placed under argon. THF (10 mL) was added and argon bubbled in the solution for 5 minutes. Then, benzyl azide (0.1 g, 0.79 mmol) and trimethylamine (0.12 mL, 0.86 mmol) were added, and the mixture was heated at 65°C overnight. The reaction was cooled to room temperature. The reaction was filtered through a plug of silica and rinsed three times with ethyl acetate (100 mL). Organic layer was washed with brine and water, dried over Na_2SO_4, filtered and solvents were removed under vacuum. Crude product was purified by SiO_2 column chromatography (PE:EtOAc, 9:1 to 3:2 (v/v)) to give compound DTS-1 (0.063 g, 96%) as a white powder.^1H NMR (300 MHz, CDCl3) δ 7.32 (dd, J = 5.0, 2.0 Hz, 6H), 7.22 (q, J = 3.6, 3.1 Hz, 10H), 7.06 – 6.99 (m, 4H), 6.23 (s, 2H), 5.27 (s, 4H). ^{13}C NMR (75 MHz, CDCl3) δ 148.28, 140.62, 134.57, 132.51, 129.89, 129.03, 128.80, 128.60, 128.05, 127.78, 123.57, 53.88. HRMS (ESI), calcd for $[M+H]^+$ at 495.2292 found 495.2293 (Δ = 0 ppm); Elemental analysis calcd for $C_{32}H_{26}N_6$: C, 77.71, H, 5.30, N, 16.99 found C, 75.35, H, 5.00, N, 15.92.

- Alternate synthesis for **DTS-1**

(1-Benzyl-1*H*-1,2,3-triazol-4-yl)phenylmethanone

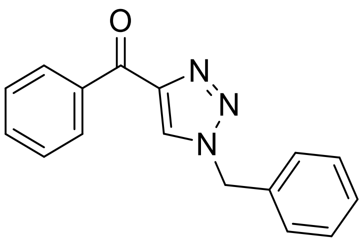

Compound was synthesized following described procedure in literature[1]. ^{1}H NMR spectrum was found in accordance with spectra reported in the literature.

DTS-1 (E/Z)

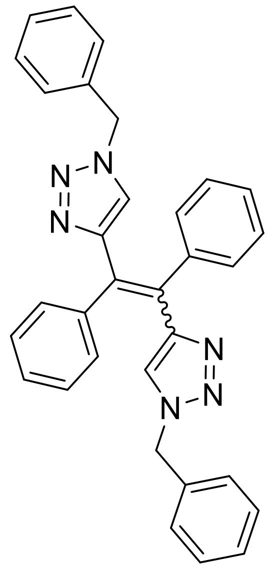

McMurry coupling was performed with (1-benzyl-1*H*-1,2,3-triazol-4-yl)phenylmethanone (0.30 g, 1.15 mmol) according to the same procedure as for **DTS-1**. After SiO_2 column chromatography (PE:EtOAc, 9:1 to 0:1 (v/v)), a mixture of E and Z isomer was obtained (0.06 g, 21%) ^{1}H NMR (300 MHz, CDCl3) δ 7.42 – 6.98 (m, 25H), 6.22 (s, 2H), 5.40 (s, 1H), 5.27 (s, 4H). ^{13}C NMR (400 MHz, CDCl3) δ 148.18, 140.59, 140.48, 134.80, 134.43, 132.38, 130.62, 129.77, 129.10, 128.93, 128.71, 128.62, 128.51, 127.96, 127.84, 127.79, 127.73, 127.70, 127.15, 123.67, 123.46, 53.92, 53.79

1-[*p*-(Diphenylamino)phenyl]-3-(triisopropylsilyl)-2-propyn-1-one

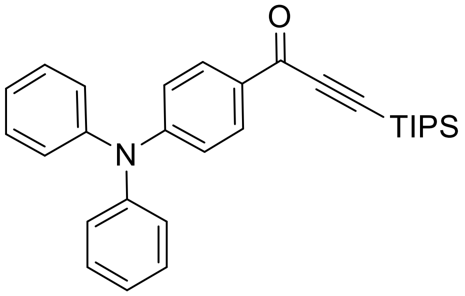

In a dry vessel, (triisopropylsilyl)acetylene (2.5 mL, 10.9 mmol, 1.5 eq.) was added to a 2.5 M solution of n-butyllithium (7 mL, 10.9 mmol, 1.5 eq.) at -80°C and was stirred for 5 minutes. Then 4-(diphenylamino)benzaldehyde (2 g, 7.3 mmol, 1 eq.) dissolved in 3 mL of THF was added to the mixture and was allowed to warm slowly to room temperature for 2 hours. After treatment with saturated aqueous ammonium chloride (50 mL) and two extractions with diethyl ether (100 mL), the combined organic layers, solvents were removed under vacuum. Crude product was used directly without further purification. The obtained yellowish oil was dissolved with 30 mL of CH_2Cl_2 and MnO_2 powder (3.2 g, 36 mmol, 5 eq.) was added. Reaction was stirred for 10h at room temperature and then filtered through a plug of silica and rinsed three times with CH_2Cl_2 (3x100 mL). Organic layer was washed with water and solvents were removed under vacuum. Crude product was purified by SiO2 column chromatography (PE only to PE: CH_2Cl_2, 4:1 (v/v)) to give product (2,58 g, 78%) as a yellow oil. ^{1}H NMR (400 MHz, CDCl3) δ 7.99 (d, J = 8.9 Hz, 2H), 7.34 (dd, J = 8.5, 7.3 Hz, 4H), 7.21 – 7.13 (m, 6H), 6.97 (d, J

= 8.9 Hz, 2H), 1.20 – 1.11 (m, 21H). ^{13}C NMR (101 MHz, CDCl3) δ 175.83, 153.25, 146.35, 131.44, 129.93, 129.90, 129.86, 129.83, 129.80, 129.63, 126.57, 126.54, 126.52, 126.48, 126.44, 126.42, 125.21, 119.17, 119.14, 119.10, 103.61, 96.39, 18.74, 11.29.

(*E*)-4,4'-Bis(diphenylamino)-α,β-bis[2-(triisopropylsilyl)ethynyl]stilbene

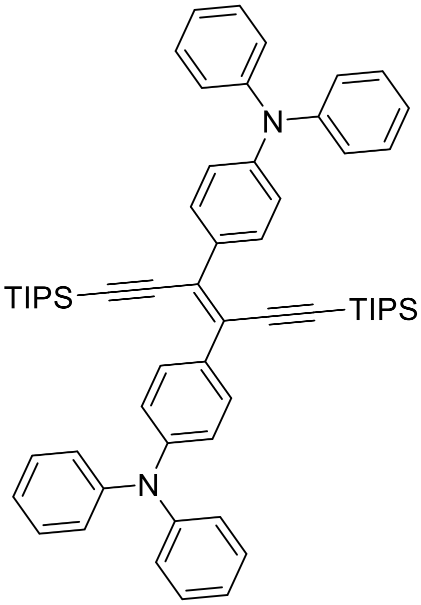

Compound was synthesized according to the same procedure as for **DTS-1**. 1-[*p*-(diphenylamino)phenyl]-3-(triisopropylsilyl)-2-propyn-1-one (1 g, 2.20 mmol) starting material afford desired product (0.490 g, 64%) as a yellow powder. ^{1}H NMR (300 MHz, CDCl3) δ 7.81 (d, J = 8.7 Hz, 4H), 7.29 – 7.22 (m, 5H), 7.17 – 7.09 (m, 9H), 7.07 – 6.97 (m, 10H), 1.02 (s, 42H). ^{13}C NMR (75 MHz, CDCl3) δ 147.80, 147.65, 133.30, 130.51, 129.38, 127.82, 124.74, 123.15, 122.50, 107.87, 101.71, 18.82, 11.54.

(E)-DTS-2

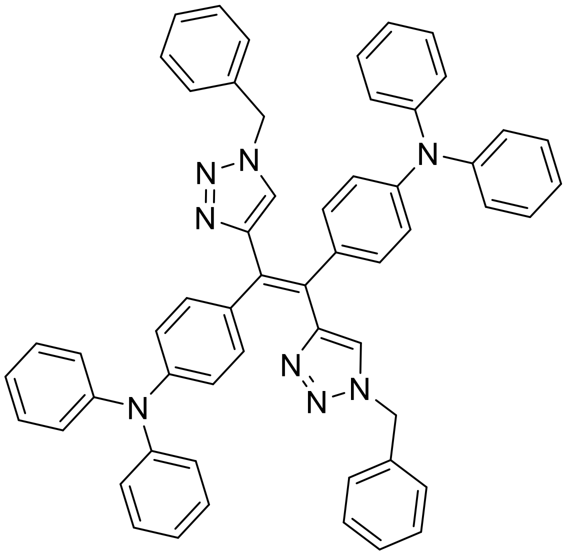

In a dry vessel, shaded from light, (*E*)-4,4'-Bis(diphenylamino)-α,β-bis[2-(triisopropylsilyl)ethynyl]stilbene (0.3 g, 0.34 mmol) was placed under argon and then solubilized in dry THF (20 mL). 1M solution of TBAF in THF (750 µL, 0.75 mmol, 2.2 eq.) was added. Reaction was

stirred for 15 min. Then, 100 mL of water were added to the vessel. Compound was extracted by washing 3 times with Et_2O (100mL). Solvent was removed under vacuum. All work-up steps were performed under red light. Crude product was used directly without further purification. The obtained brown powder was dissolved in CH_2Cl_2 (15 mL) and benzyl azide (136 µL, 1.00 mmol, 3 eq.) was added. Argon was bubbled in the solution for 15 min. In another vessel, copper sulfate (0.19 g, 0.75 mmol, 2.2 eq.) and sodium L-ascorbate (0.15 g, 0.75 mmol, 2.2 eq.) were dissolved in water (15 mL). Argon was bubbled in the solution for 15 min. The aqueous solution was then added to the organic reaction mixture. The reaction was stirred protected from light overnight. Then, 200 mL of CH_2Cl_2 was added to the reaction mixture. Organic phase was washed 3 times with water (200 mL) then with, dried over $NaSO_4$, filtered and solvent was removed under vacuum. All work-up steps were performed under red light. Crude product was purified through precipitation in cyclohexane to give compound **(E)-DTS-2** (204 mg, 70%) as a pale brown powder. ^1H NMR (400 MHz, CDCl3) δ 7.27 – 7.13 (m, 14H), 7.08 – 6.90 (m, 20H), 6.82 (d, J = 8.6 Hz, 4H), 6.66 (s, 2H), 5.34 (s, 4H). ^{13}C NMR (101 MHz, CDCl3) δ 149.04, 147.58, 147.11, 134.97, 134.44, 131.73, 131.06, 129.39, 129.19, 128.74, 127.54, 124.87, 124.00, 123.24, 122.80, 53.96. HRMS (ESI), calcd for M+. at 828.3683 found 828.3681 (Δ = 0 ppm); Elemental analysis calcd for C56H44N8: C, 81.13, H, 5.35, N, 13.52 found C, 78.80, H, 5.29, N, 12.38.

(Z)-DTS-2

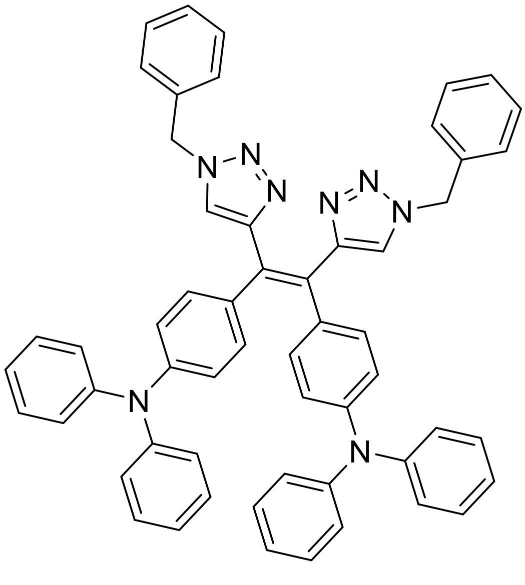

(E)-DTS-2 (30 mg, 0.036 mmol) was dissolved in CH_2Cl_2 (20 mL) and stirred overnight under LED irradiation at λ=365 nm, P=5mW/cm^2 in a photoreactor PhotoRedOx Box from HepatoChem. Solvent was evaporated under vacuum. Diastereomeric mixture was separated on neutral alumina column chromatography (CH_2Cl_2: EtOAc, 1:0 to 3:2 (v/v)) and (**Z)-DTS-2** was obtained as a yellow green powder (9.5 mg, 31%). ^1H NMR (400 MHz, CDCl3) δ 7.39 – 7.31 (m, 3H), 7.19 (dtd, J = 14.6, 7.2, 2.0 Hz, 6H), 7.12 (s, 1H), 7.05 (d, J = 7.4 Hz, 4H), 6.99 (t, J = 7.3 Hz, 2H), 6.92 (d, J = 8.7 Hz, 2H), 6.82 (d, J = 8.7 Hz, 2H), 5.38 (s, 2H); HRMS (ESI), calcd for M+. at 828.3683 found 828.3690 (Δ = -0.8 ppm).

(E)-DTS-Pyr

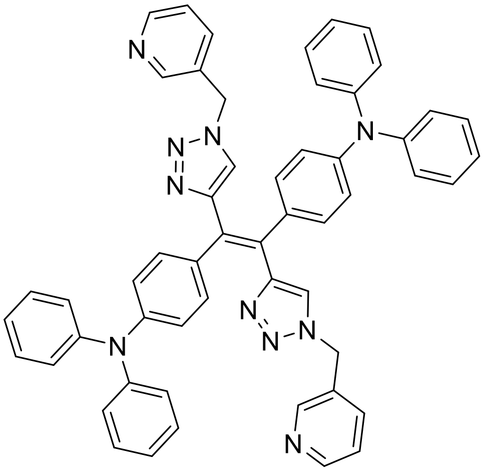

Compound **DTS-Pyr** was synthetized following the same procedure as **(E)-DTS-2** by reaction of **(*E*)-4,4'-Bis(diphenylamino)-α,β-bis[2-(triisopropylsilyl)ethynyl]stilbene** (30mg, 34 µmol) and TBAF 1M in THF (75 µL, 75 µmol, 2.2 eq.). Then, the crude product (34 µmol) was put to react with 3-azidomethylpyridine 0.5M THF (100µL, 100 µmol, 3 eq.), copper sulfate (0.19 g, 0.75 mmol, 2.2 eq.) and sodium L-ascorbate (0.15 g, 0.75 mmol, 2.2 eq.). Pure product was obtained after precipitation in mixture of CH_2Cl_2 and cyclohexane as a yellow solid (18 mg, 63%). ^{1}H NMR (400 MHz, CD2Cl2) δ 8.62 (s, 2H), 7.40 (d, J = 7.7 Hz, 1H), 7.34 – 7.18 (m, 5H), 7.11 – 7.01 (m, 6H), 7.00 (d, J = 8.6 Hz, 2H), 6.96 – 6.87 (m, 2H), 6.80 (s, 1H), 5.44 (s, 2H). ^{13}C NMR (101 MHz, CD2Cl2) δ 150.33, 149.32, 149.04, 147.78, 147.53, 135.23, 134.71, 131.90, 131.27, 129.73, 125.22, 124.93, 124.37, 123.73, 123.69, 123.44, 122.66, 51.72. HRMS (ESI), calcd for M+H. at 831.3667 found 831.3668 (Δ = -0.2 ppm).

(E)-DTS-OH

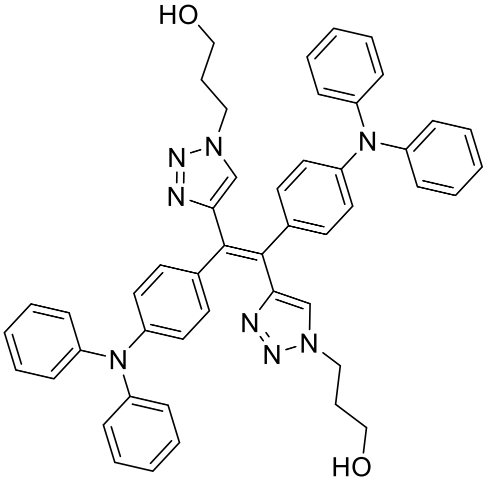

Compound **DTS-OH** was synthetized following the same procedure as **(E)-DTS-2**. Compound **DTS-Pyr** was synthetized following the same procedure as **(E)-DTS-2** by reaction of **(*E*)-4,4'-Bis(diphenylamino)-α,β-bis[2-(triisopropylsilyl)ethynyl]stilbene** (30mg, 34 µmol) and TBAF 1M in THF (75 µL, 75 µmol, 2.2 eq.). Then, the crude product (34 µmol) was put to react with 3-Azido-1-propanol (9µL, 100 µmol, 3 eq.), copper sulfate (0.19 g, 0.75 mmol, 2.2 eq.) and sodium L-ascorbate

(0.15 g, 0.75 mmol, 2.2 eq.). Pure product was obtained after precipitation in mixture of CH_2Cl_2 and pentane as a brown solid (21 mg, 43%). ^{1}H NMR (300 MHz, CDCl3) δ 7.26 (t, J = 15.3 Hz, 3H), 7.17 – 6.94 (m, 11H), 6.78 (s, 1H), 4.40 (t, J = 6.6 Hz, 2H), 3.54 (dd, J = 5.2 Hz, 2H), 2.02 (p, J = 6.2 Hz, 2H). ^{13}C NMR (101 MHz, CD2Cl2) δ 148.64, 147.91, 147.55, 135.31, 131.93, 131.45, 129.72, 125.07, 124.61, 123.60, 123.12, 59.01, 47.04, 33.01. HRMS (ESI), calcd for M+H. at 765.3652 found 765.3660 (Δ = 1.0 ppm).

Compound characterization

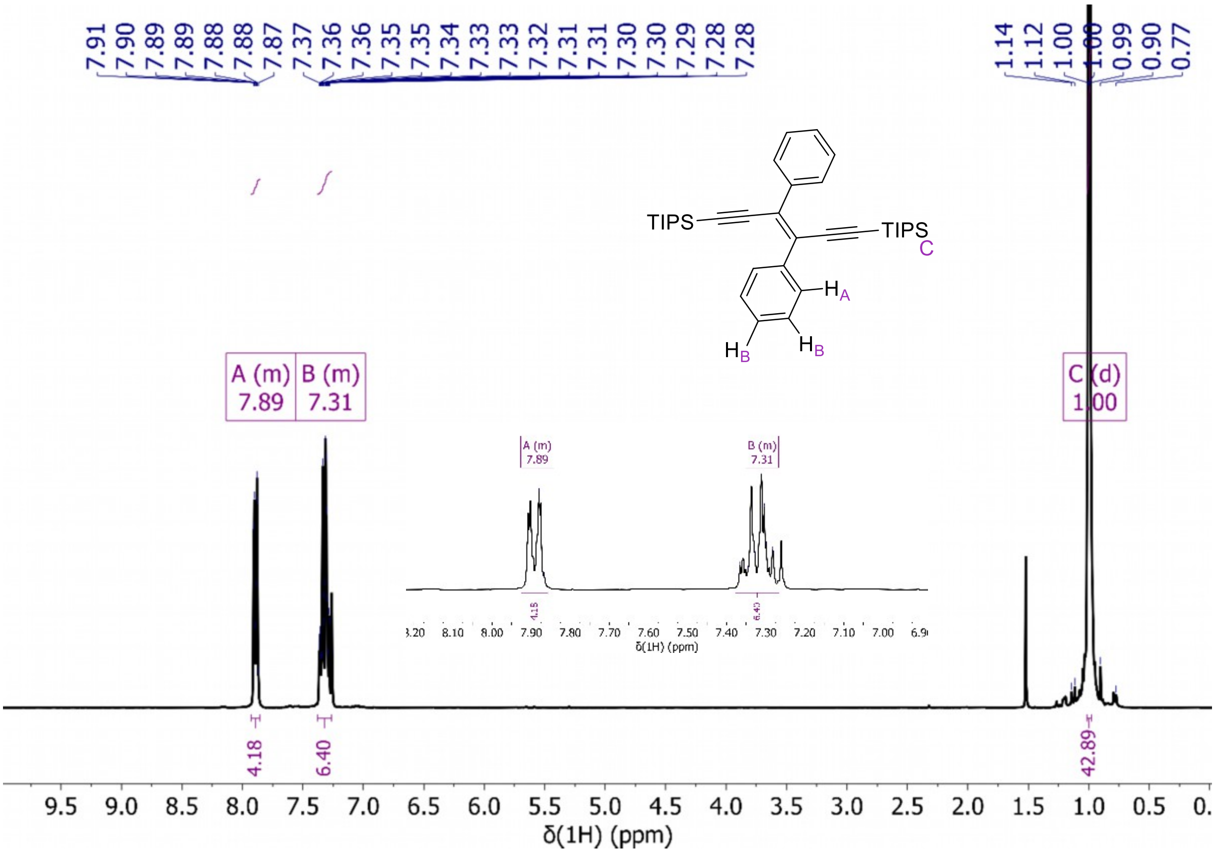

Figure S1: ¹H NMR spectrum of (E)-[(triisopropylsilyl)ethynyl]stilbene in $CDCl_3$

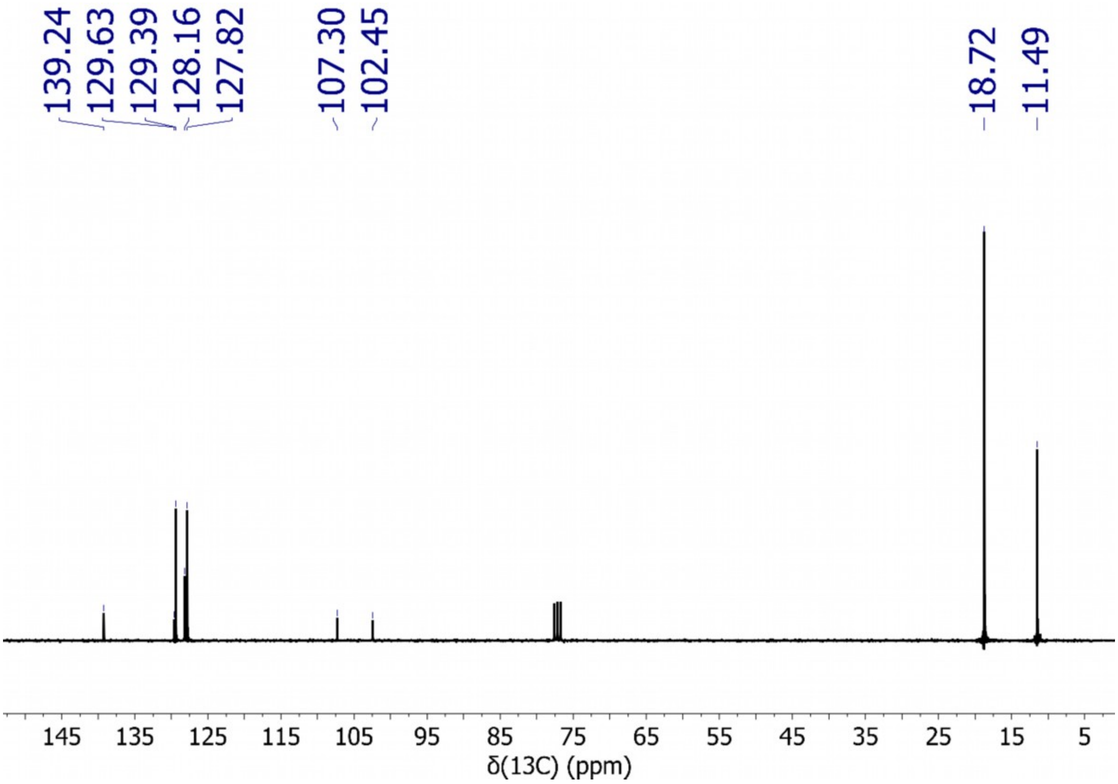

Figure S2: ¹³C NMR spectrum of (E)-[(triisopropylsilyl)ethynyl]stilbene in $CDCl_3$

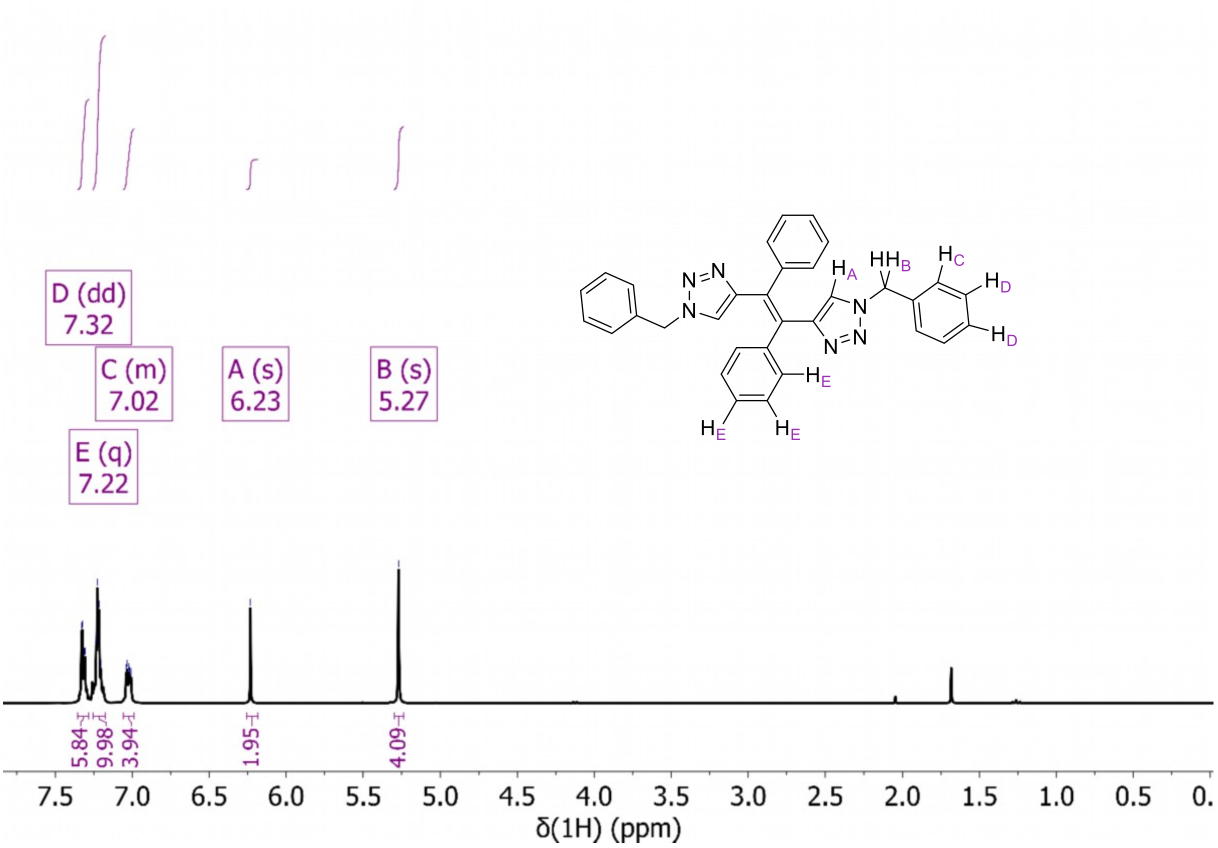

Figure S3: ^{1}H NMR spectrum of (E)-DTS-1 in $CDCl_3$

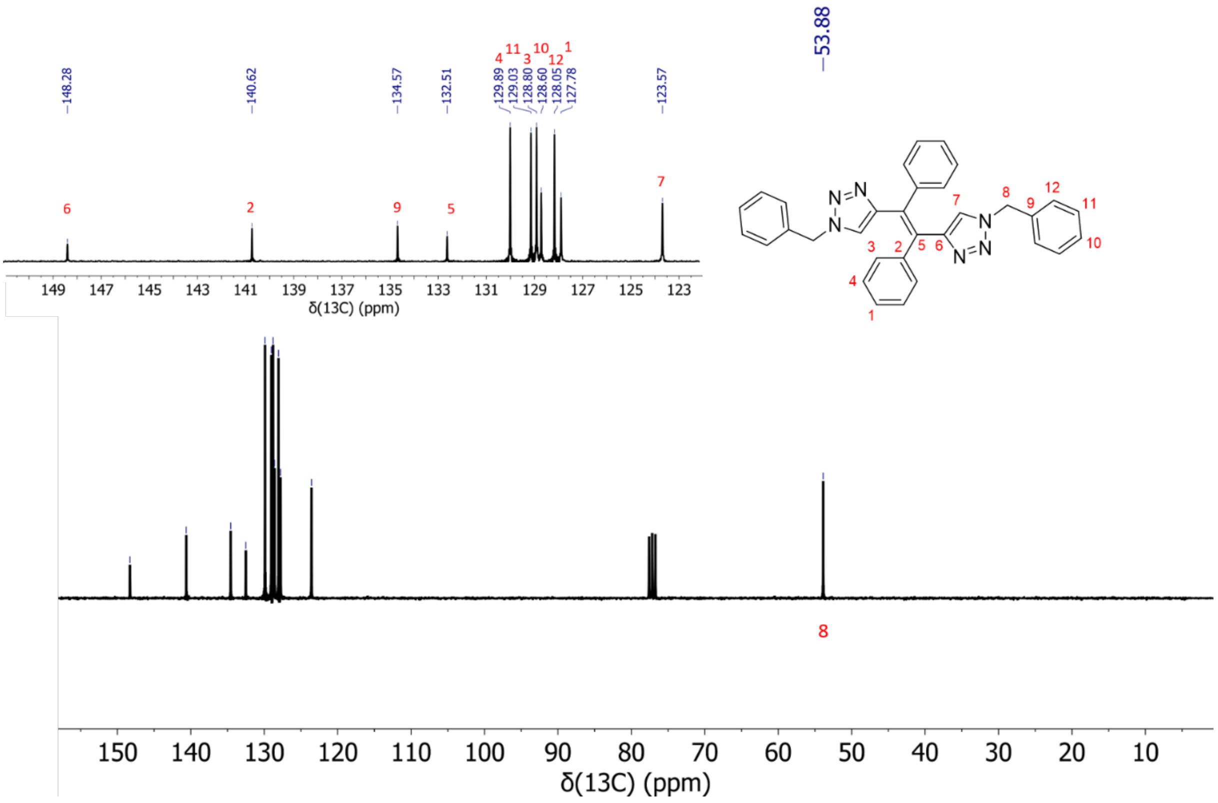

Figure S4: ^{13}C NMR spectrum of (E)-DTS-1 in $CDCl_3$

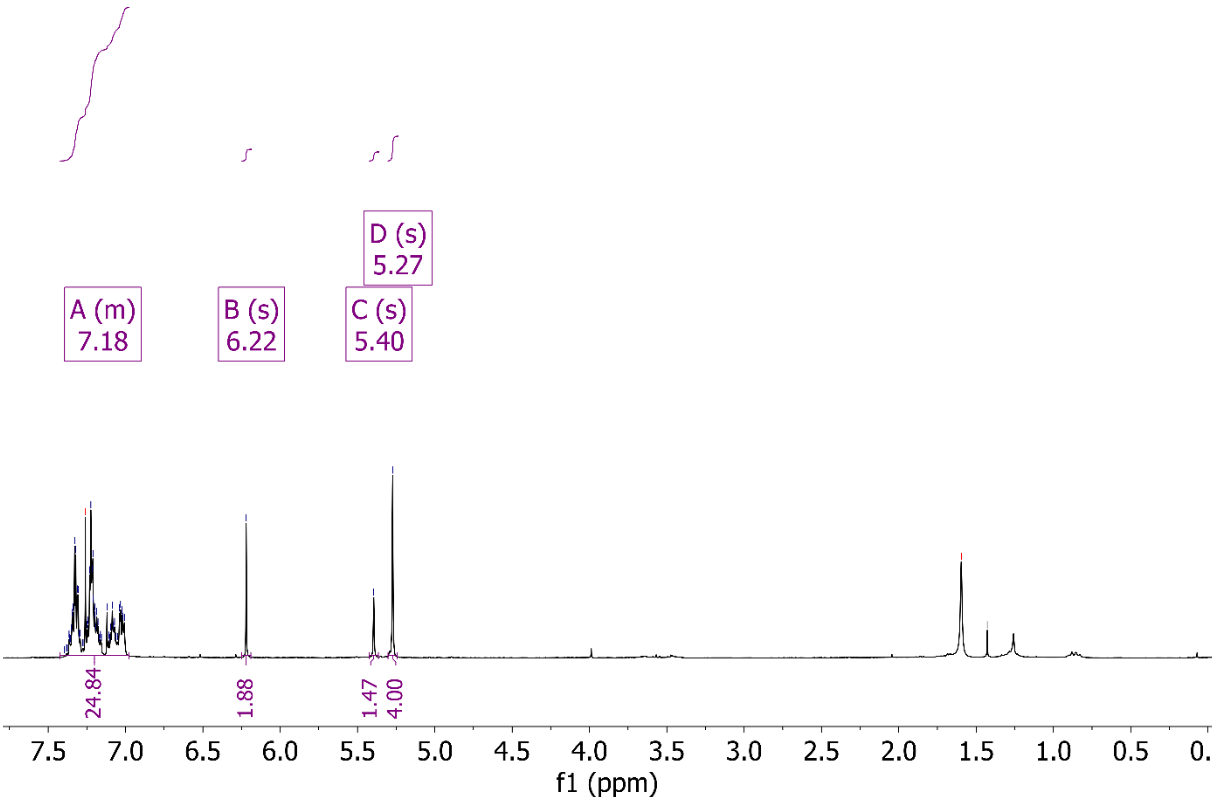

Figure S5: ^{1}H NMR spectrum of the mixture of (E)-DTS-1 and (Z)-DTS-1 in $CDCl_3$

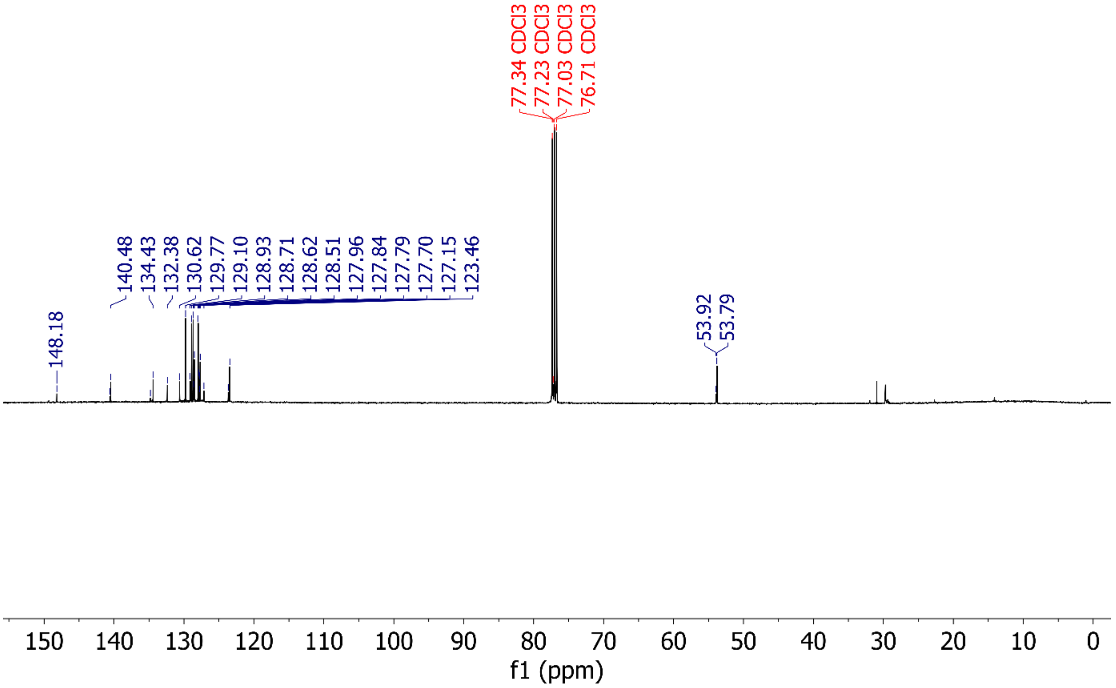

Figure S6: ^{13}C NMR spectrum of the mixture of (E)-DTS-1 and (Z)-DTS-1 in $CDCl_3$

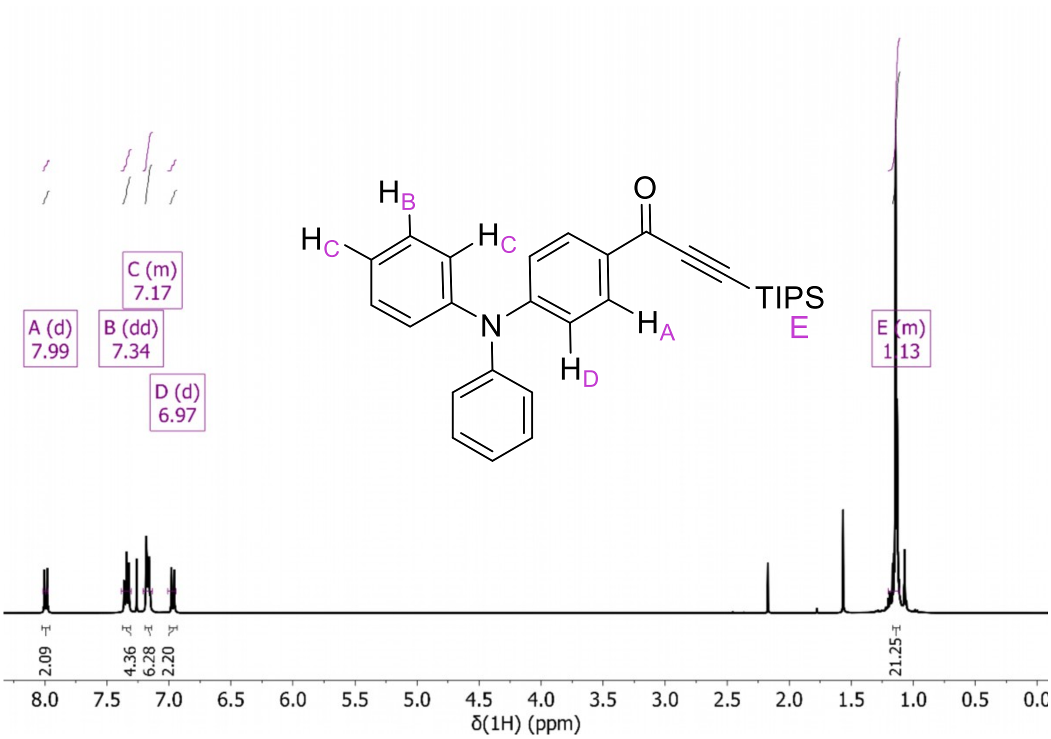

*Figure S7: ^{1}H NMR spectrum of 1-[*p*-(diphenylamino)phenyl]-3-(triisopropylsilyl)-2-propyn-1-one in $CDCl_3$*

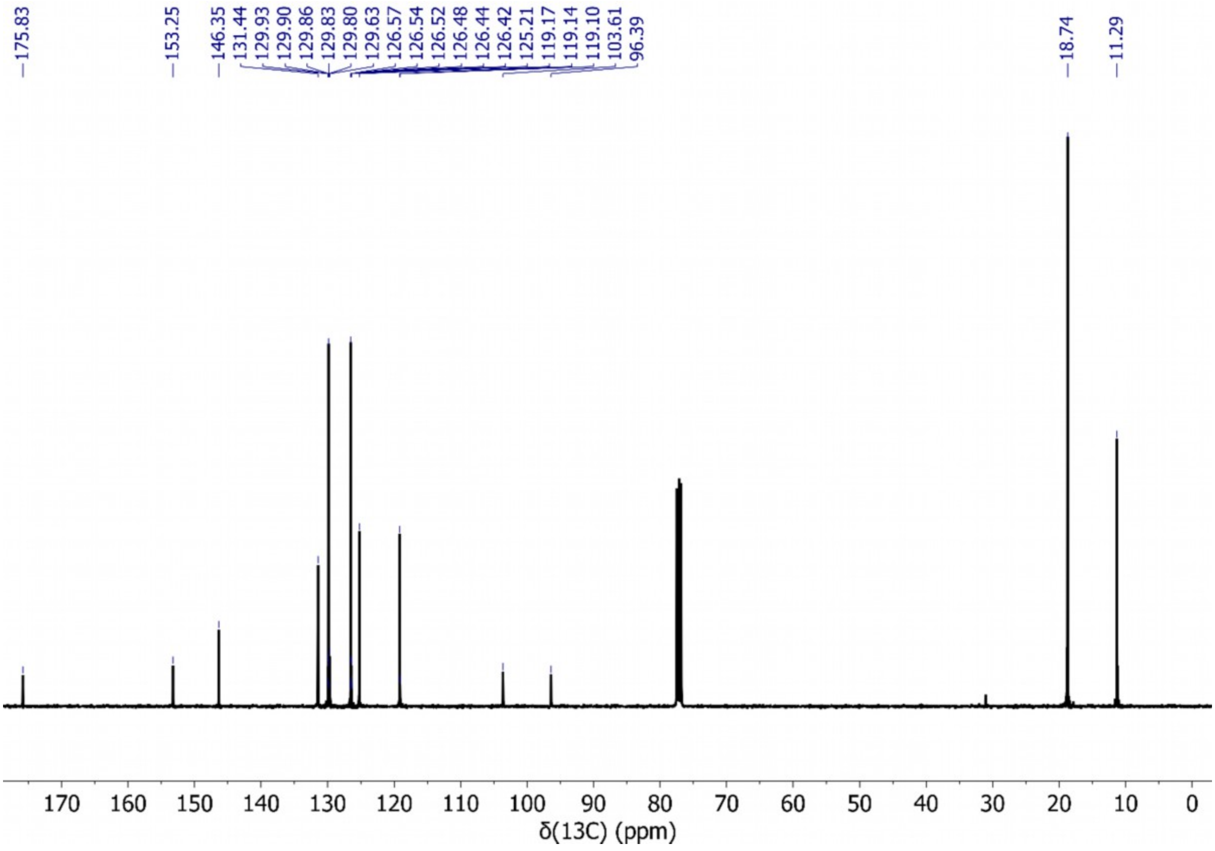

*Figure S8 : ^{13}C NMR spectrum of 1-[*p*-(diphenylamino)phenyl]-3-(triisopropylsilyl)-2-propyn-1-one in $CDCl_3$*

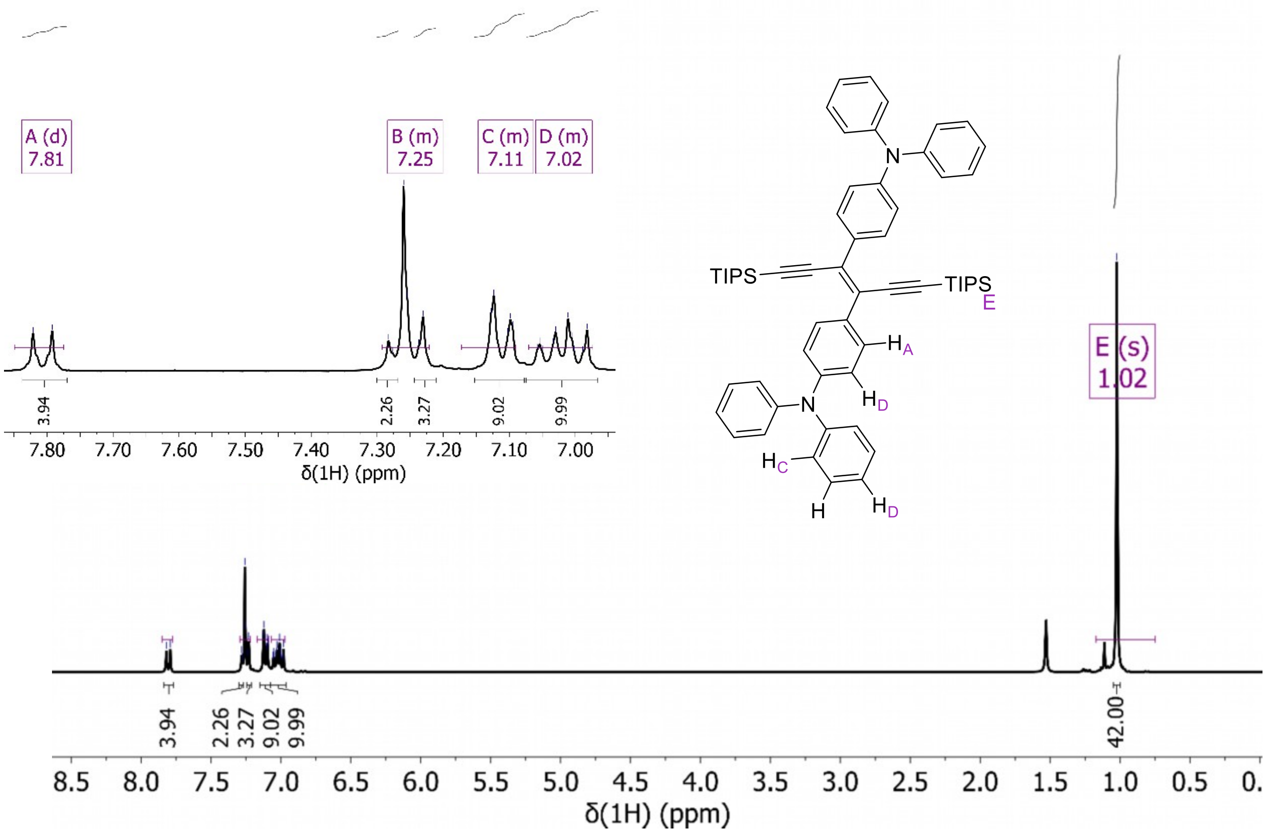

Figure S9: ^{1}H NMR spectrum of (E)-4,4'-Bis(diphenylamino)-α,β-bis[2-(triisopropylsilyl)ethynyl]stilbene in $CDCl_3$

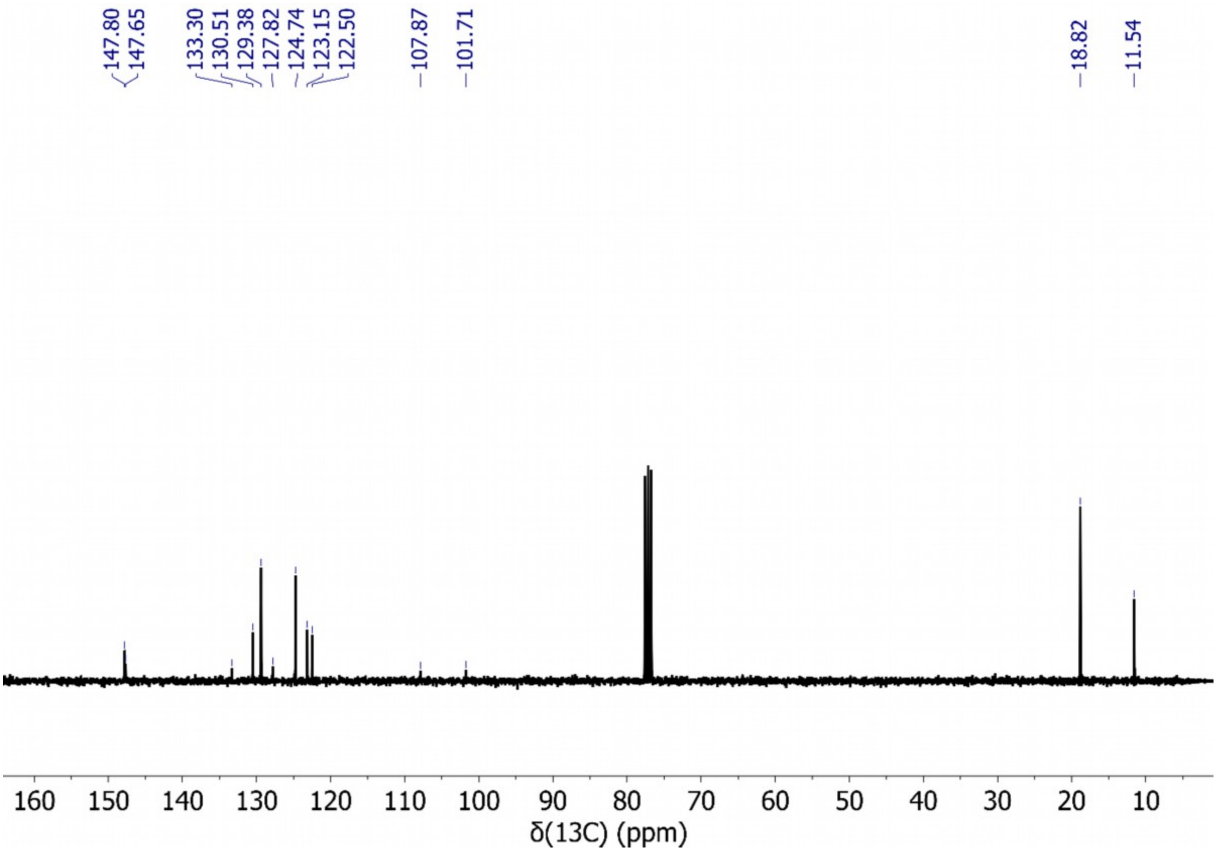

Figure S10: ^{13}C NMR spectrum of (E)-4,4'-bis(diphenylamino)-α,β-bis[2-(triisopropylsilyl)ethynyl]stilbene in $CDCl_3$

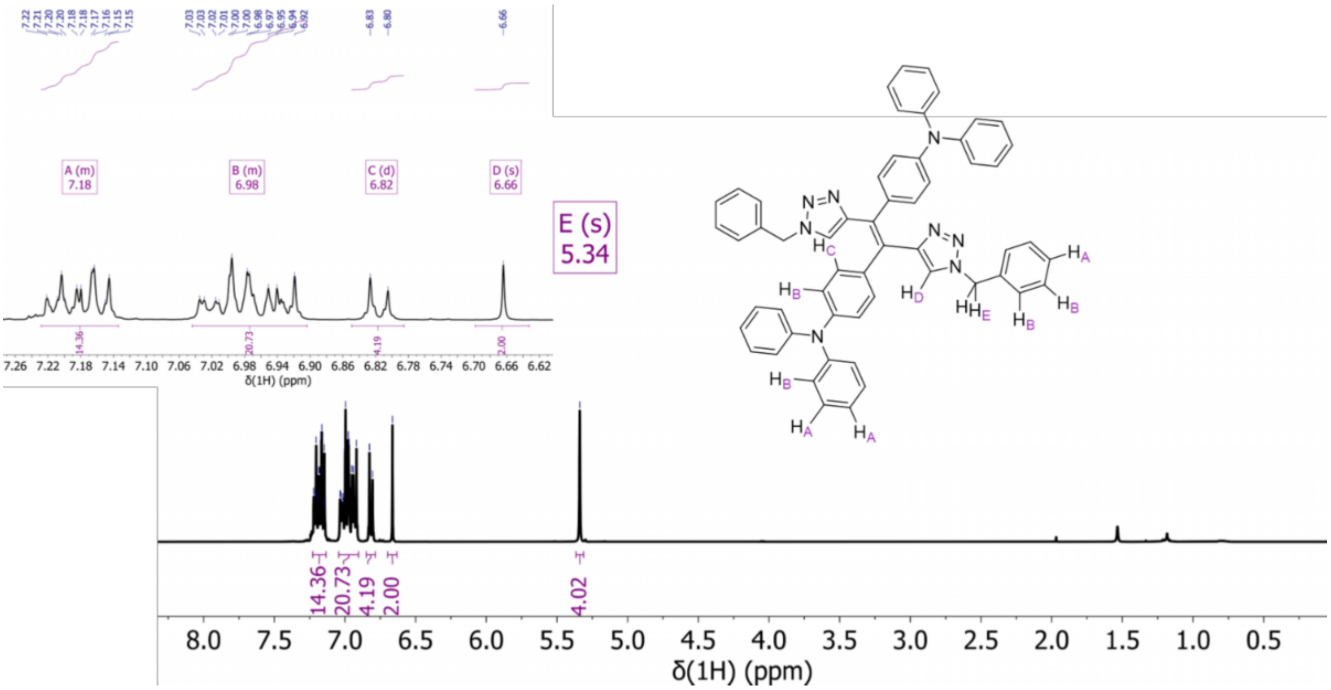

Figure S11: ^{1}H NMR spectrum of (E)-DTS-2 in $CDCl_3$

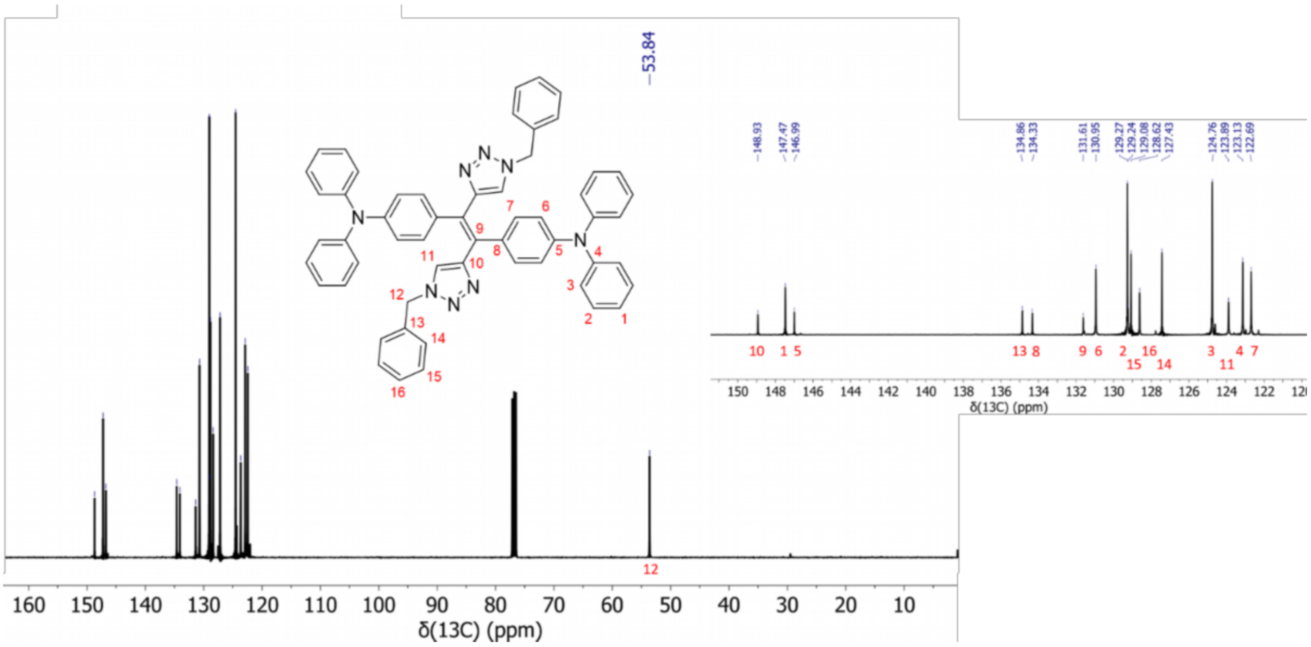

Figure S12: ^{13}C NMR spectrum of (E)-DTS-2 in $CDCl_3$

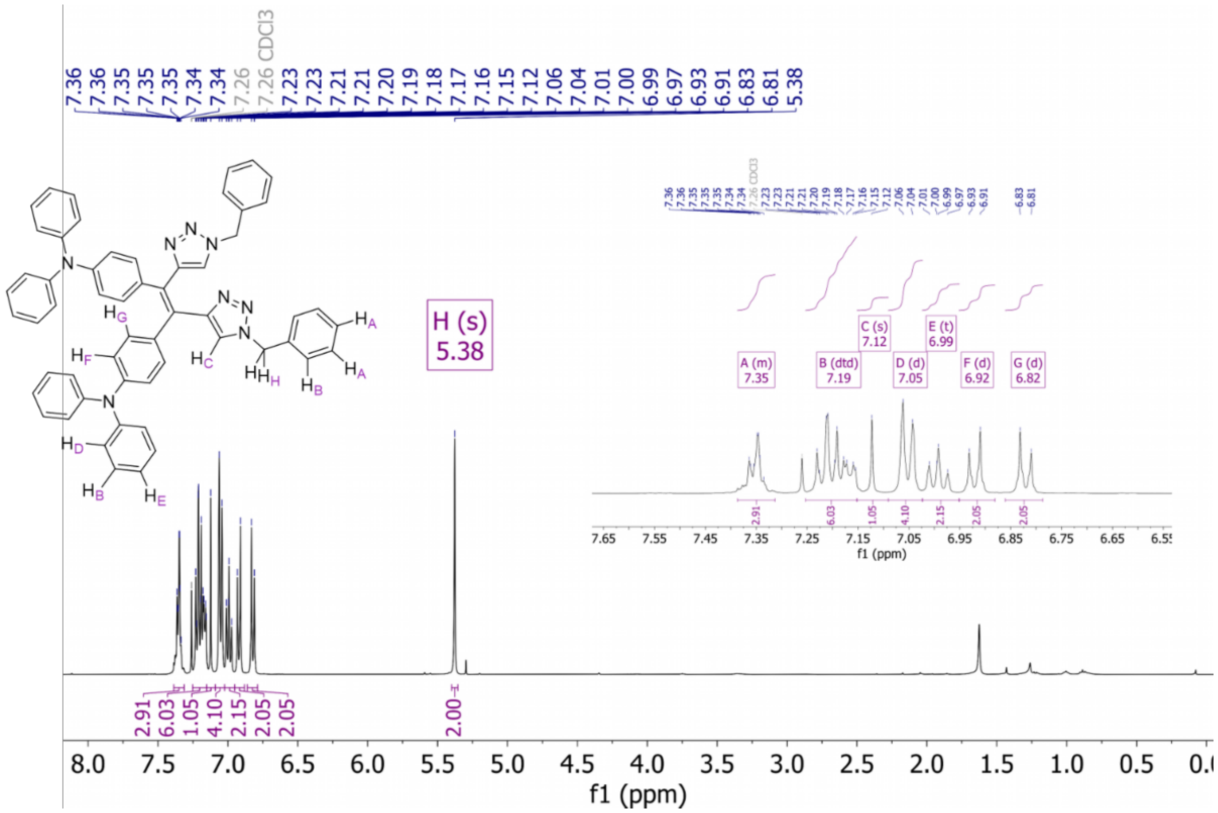

Figure S13: ^{1}H NMR spectrum of (Z)-DTS-2 in $CDCl_3$

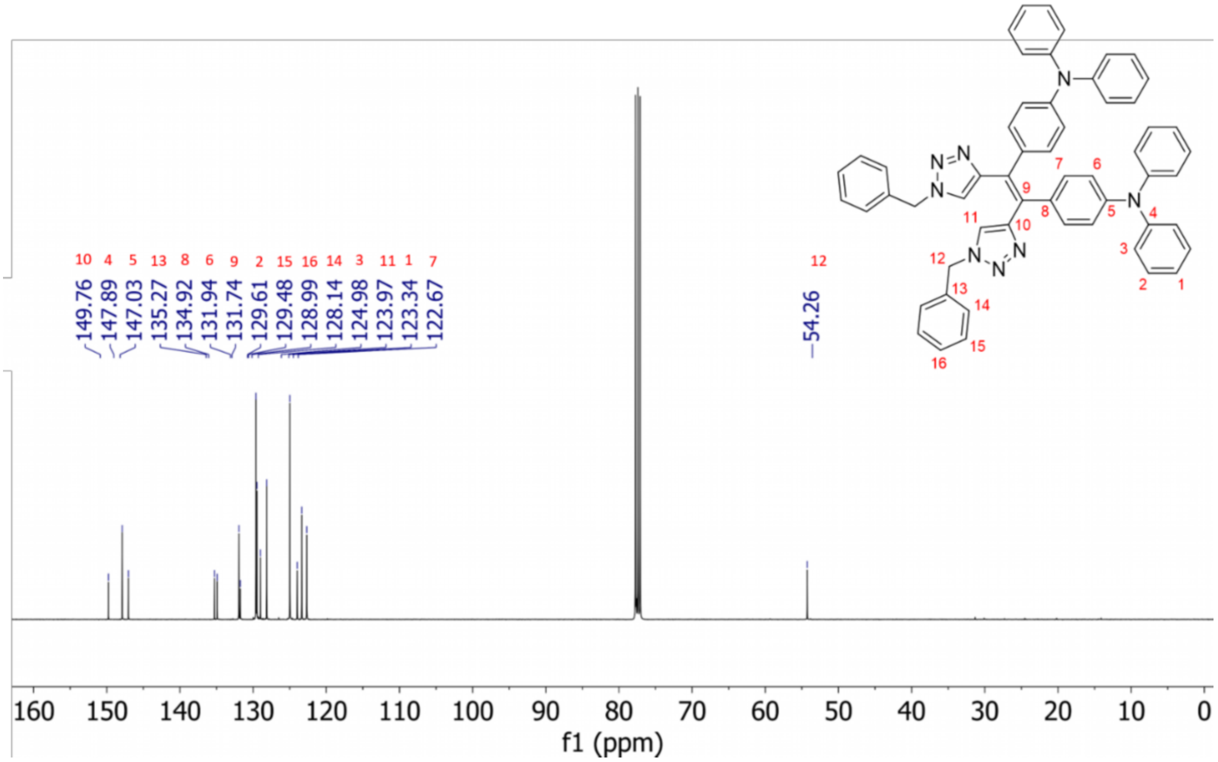

Figure S14: ^{1}H NMR spectrum of (Z)-DTS-2 in $CDCl_3$

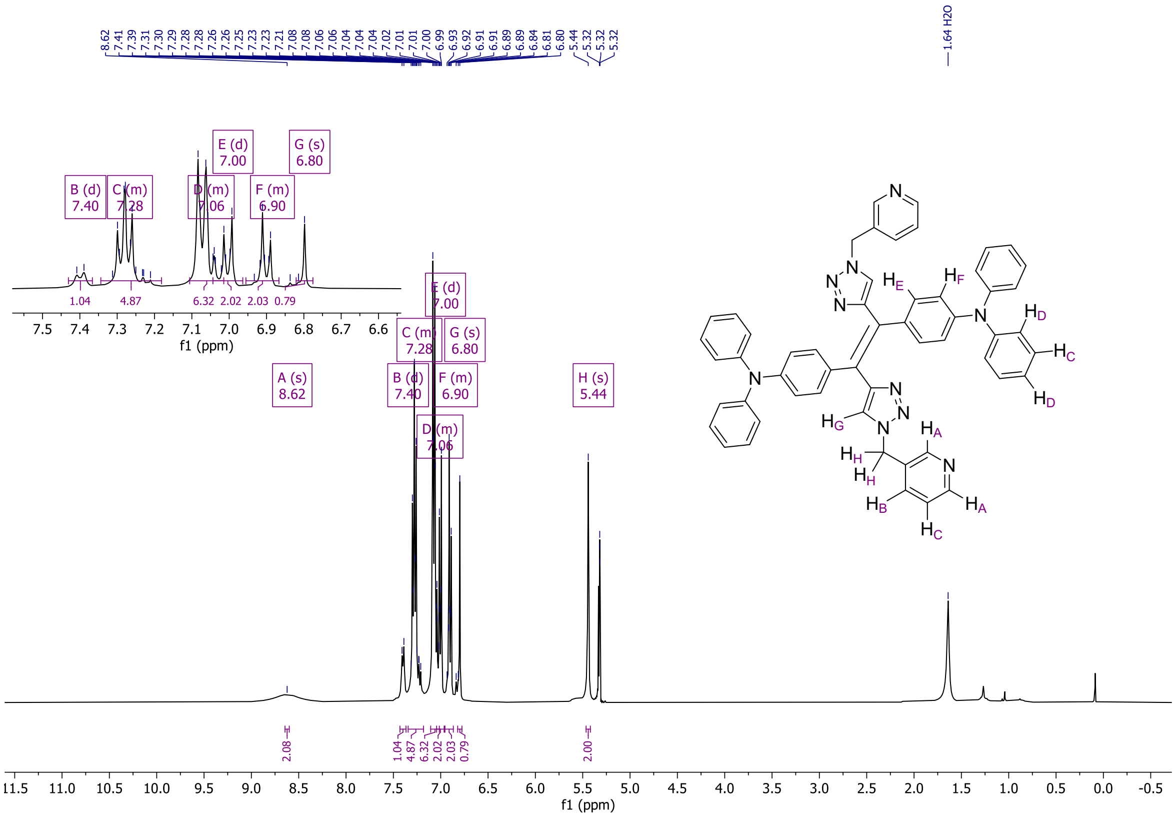

Figure S15: ^{1}H NMR spectrum of (E)-DTS-Pyr in CD_2Cl_2.

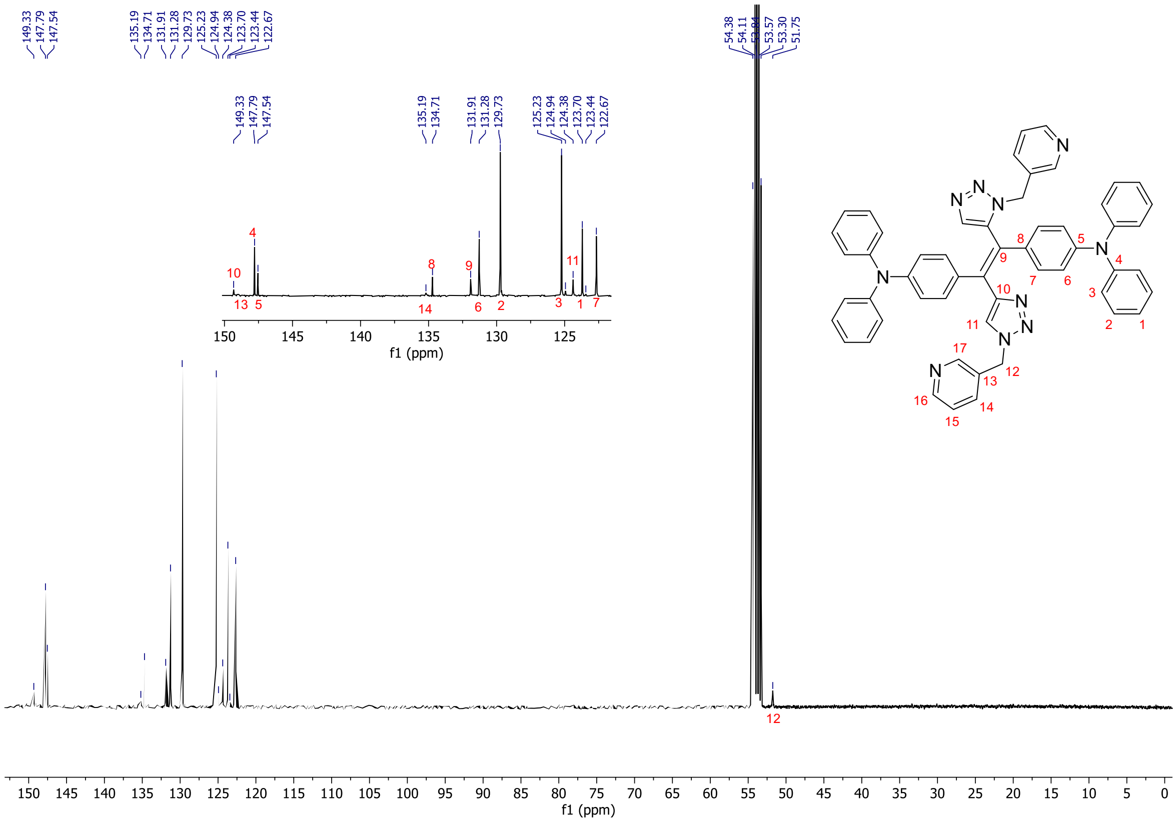

Figure S16: ^{13}C NMR spectrum of (E)-DTS-Pyr in CD_2Cl_2.

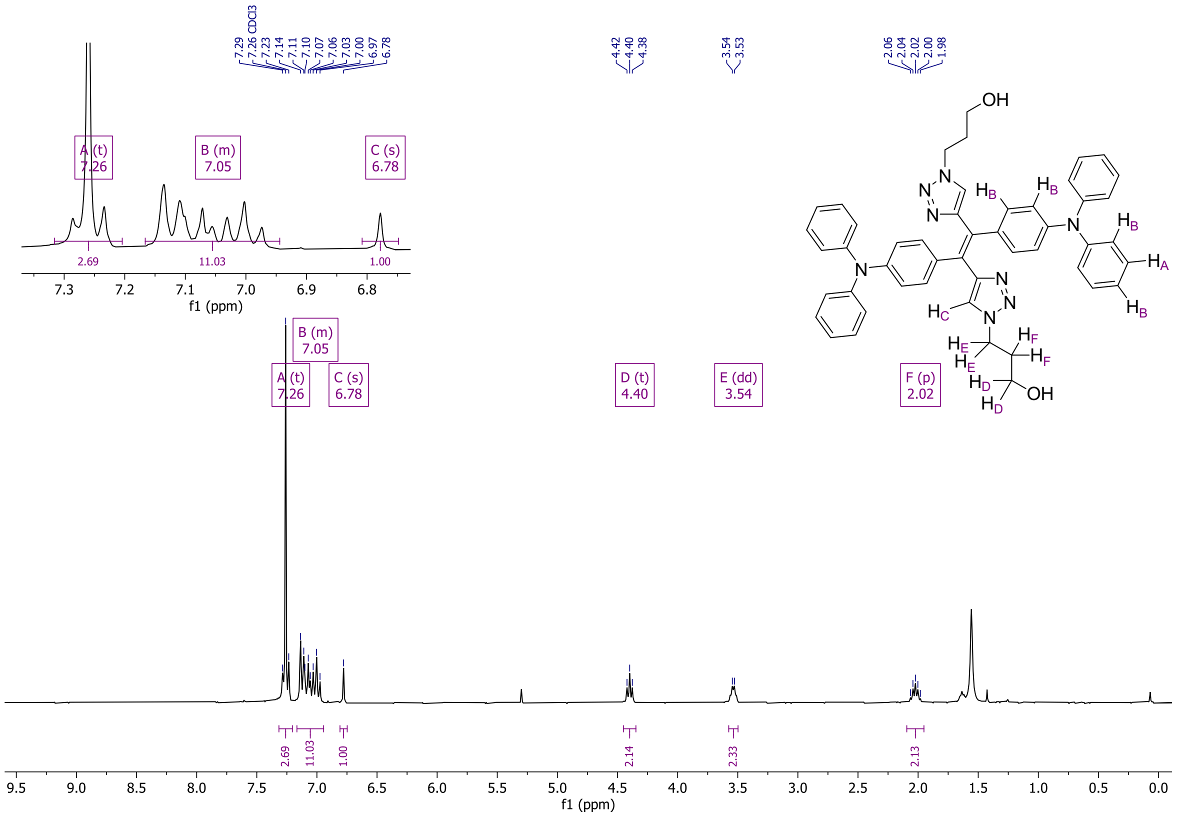

Figure S17: ¹H NMR spectrum of (E)-DTS-OH in $CDCl_3$

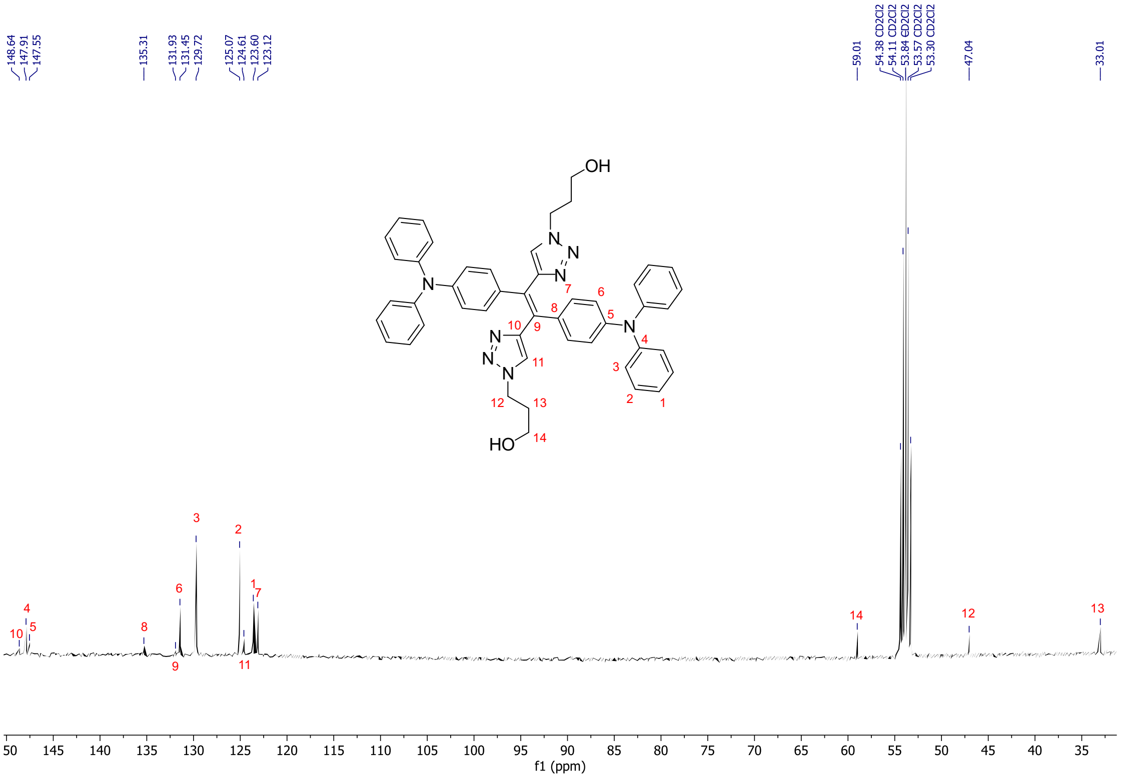

Figure S18: ¹³C NMR spectrum of (E)-DTS-OH in CD_2Cl_2

Single crystal X-ray diffraction

Single crystals were grown from by slow diffusion of cyclohexane into dichloromethane. A suitable crystal was selected and mounted on a Xcalibur, Atlas, Gemini ultra diffractometer for compound **(E)-DTS-1** or mounted on a MITIGEN holder in perfluoroether oil on a XtaLAB Synergy, Dualflex, HyPix-Arc 100 diffractometer for **(E)-DTS-2**. The structure was solved with the ShelXT (Sheldrick, 2015) solution program using dual methods and by using Olex2 (Dolomanov et al., 2009) as the graphical interface. The model was refined with ShelXL 2018/3 (Sheldrick, 2015) using full matrix least squares minimisation on F^2.

Table S1: Crystallographic parameters

**Compound**	**(E)-dts-1**	**(E)-DTS-2**
Formula	$C_{32}H_{26}N_6$	$C_{68}H_{68}N_8$
$D_{calc.}$/ g cm^{-3}	1.261	1.248
m/mm^{-1}	0.077	0.568
Formula Weight	494.59	997.30
Colour	colourless	yellow
Shape	block	plate-shaped
Size/mm^3	0.55×0.50×0.24	0.23×0.15×0.07
T/K	150.01(10)	99.9(2)
Crystal System	orthorhombic	triclinic
Space Group	*Pbca*	*P*-1
a/Å	9.0736(12)	7.77940(10)
b/Å	14.8909(18)	9.85000(10)
c/Å	19.280(2)	18.6746(2)
$a/^{\circ}$	90	78.9860(10)
$b/^{\circ}$	90	81.9550(10)
$g/^{\circ}$	90	71.4680(10)
V/Å^3	2605.0(5)	1326.95(3)
Z	4	1
Z'	0.5	0.5
Wavelength/Å	0.71073	1.54184
Radiation type	Mo K_a	Cu K_a
$Q_{min}/^{\circ}$	2.833	4.794
$Q_{max}/^{\circ}$	29.632	78.304
Measured Refl's.	17447	49438
Indep't Refl's	3323	5300
Refl's I≥2 s(I)	2326	4972
R_{int}	0.0669	0.0252
Parameters	173	344
Restraints	0	0
Largest Peak	0.347	0.658
Deepest Hole	-0.360	-0.371
GooF	1.088	1.041
wR_2 (all data)	0.2028	0.1223
wR_2	0.1605	0.1206
R_1 (all data)	0.1019	0.0481
R_1	0.0669	0.0461

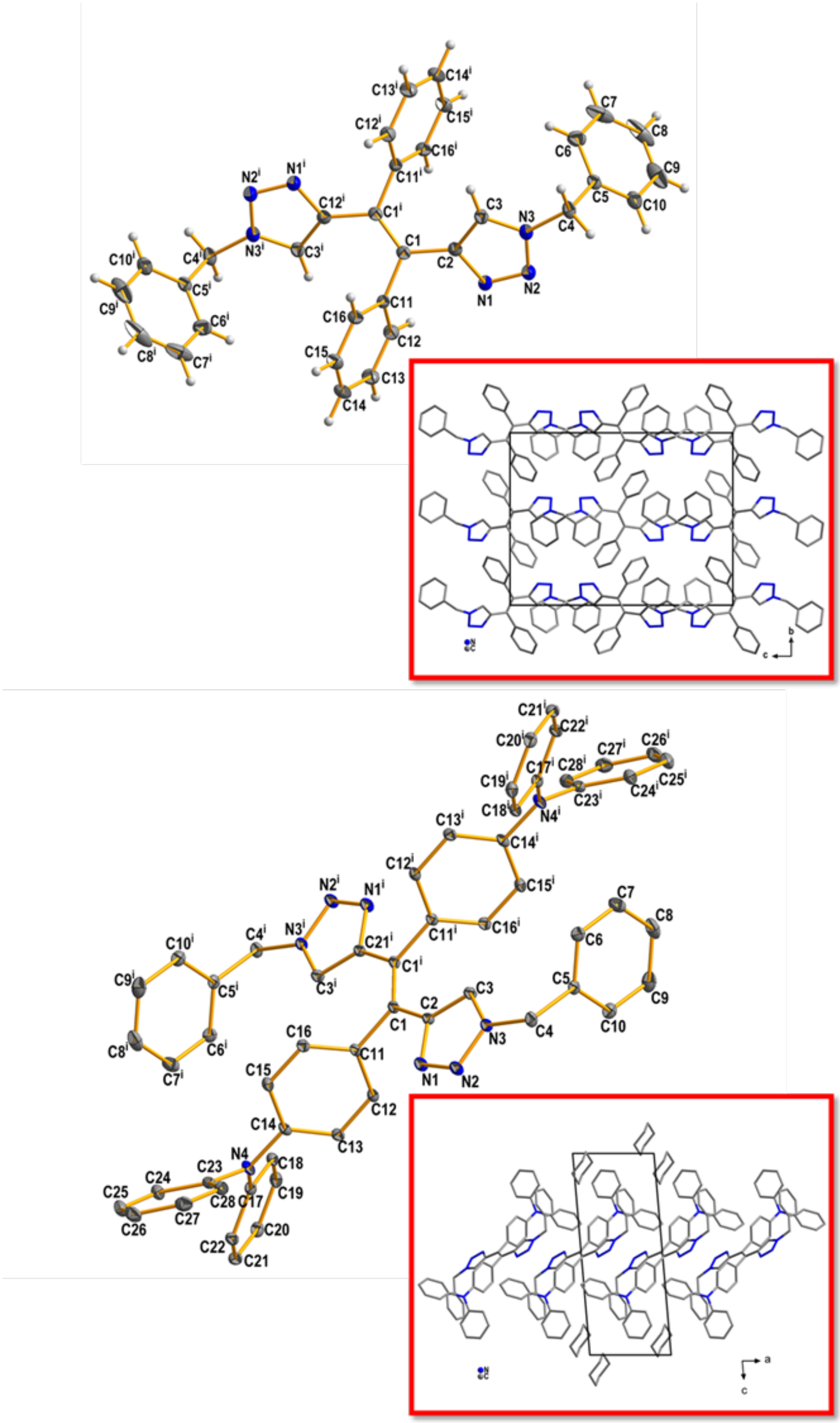

Figure S19: Molecular views of ***(E)-DTS-1*** *(top) and* ***(E)-DTS-2*** *(bottom) with displacement ellipsoids plotted at 30 % probability level (symmetry: (i) 1-x, 1-y, 1-z; Unit cells and packing diagram of* ***(E)-DTS-1*** *(red frame, top) and* ***(E)-DTS-2*** *(red frame bottom) ; Hydrogen atoms were omitted for clarity.*

Table S1: Values of some relevant bond distance and dihedral angles measured from the crystallogaphic structure. Labelling of atom refers to numbering used in Fig. S19.

geometric parameter	**(E)-DTS-1**
d(C1-C1^{i})	1.356 Å
D(C2-C1- C1^{i}-C12^{i})	180°
D(C12-C11-C1- C2)	71.6°

geometric parameter	**(E)-DTS-2**
d(C1-C1^{i})	1.355 Å
D(C2-C1- C1^{i}-C21^{i})	180°
D(C16-C11-C1- C2)	105.4°
D(C15-C14-N4C23)	47°

Photophysical properties

All measurement were performed using spectroscopic grade solvents. Absorption spectra (UV-Vis) were recorded on a dual beam Jasco 670 spectrometer and fluorescence spectra in solutions were recorded on a Horiba Jobin-Yvon Fluorolog-3® spectrofluorimeter. Spectra were reference corrected for both the excitation source light intensity variation (lamp and grating) and the emission spectral response (detector and grating).

Luminescence quantum yields Φ_f were measured in diluted solutions with an absorbance lower than 0.1, by using the following Equation 1:

$$\frac{\Phi_{fx}}{\Phi_{fr}} = \frac{A_r(\lambda)}{A_x(\lambda)} \frac{n_x^2}{n_r^2} \frac{Dx}{Dr}$$

where A(λ) is the absorbance (or optical density) at the excitation wavelength, n the refractive index of the solvent and D the integrated luminescence intensity. "r" and "x" stand for reference and sample, respectively. Here, the reference is quinine sulphate in 0.5 M H_2SO_4 (Φ_{fr} = 0.55). Excitations of reference and sample compounds were performed at the same wavelength. The reported results are the average of 4 independent measurements at various absorbances (comprised between 0.01–0.1) for both sample and reference. The plot of the integrated luminescence intensity vs. absorbance gives straight line with good correlation coefficients and the slope S can be determined for both sample (x) and reference (r).

Sample for nanoprecipitation experiment were prepared from a concentrated solution in DMSO mixed with relevant proportion of DMSO and water in ultrasound stirring bath.

Absolute fluorescence quantum yields in solid Φ_S were measured using a calibrated integrative sphere collecting all the emission (2π steradians covered with spectralon®, model G8 from GMPF. Four measurements were made with constant excitation and emission to give four integrated intensities. E_c and E_a are the integrated fluorescence spectra of direct excitation with and without the sample. L_c and L_a are the integrated excitation spectra of the sphere with and without the sample. A neutral density filter (transmittance = 0.5%) was used to reduce the intensity of the excitation profile. Quantum yields are given by Equation 2:

$$\Phi_s = \frac{E_c - E_a}{L_a - L_c}$$

Irradiation experiments were performed in integrative sphere (model G6 from GMPF) collecting all the emission from a focused 300 or 365 LED. Power at the focal point was measured with a power-meter equipped with a S120VC photodiode. Irradiated sample had an absorbance coefficient of above 2 so all the measured photon flux is evaluated to be fully absorbed by the sample. Photoisomerization quantum yield $\Phi_{E\to Z}$ are calculated from quantity of Z isomer in sample (quantified from NMR spectra integration) as a function of time. Tangent method is used, approximating that as the Z isomer does not absorb any light in the first instant of the irradiation experiment. $\Phi_{E\to Z}$ can then be calculated as Equation 3:

$$\Phi_{E\to Z} = \frac{N_{E\to Z}}{N_{photon}}$$

With N_{photon} the number of photons emitted per secund

$$N_{photon} = \frac{P\lambda}{hc}$$

and $N_{E\to Z}$ the number of molecules isomerized per secund (i.e. slope of the tangent), λ the irradiation wavelength, h the planck constant and c the celerity of light.

Images of crystalline powders have been with an Olympus BX53M microscope (4x zoom) under ambient light or UV light from a 365 nm LED. The XRD pattern of powders were measured using a Malvern Panalytical Empyrean X-ray diffractometer.

Photoisomerization quantum yield measurements

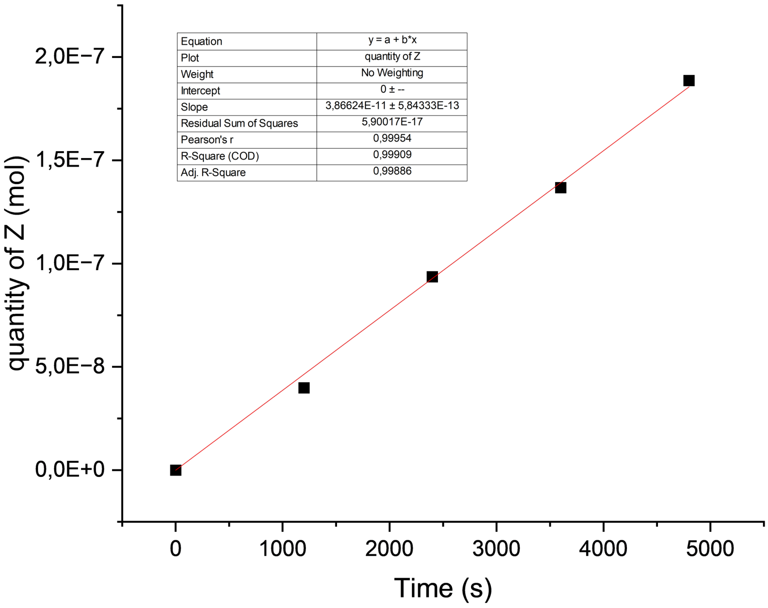

*Figure S20: Linear fit of the quantity of (Z)-isomer as a function of time obtained by irradiation of a **(E)-DTS-1** sample at 300 nm with a power of 0.52 mW.*

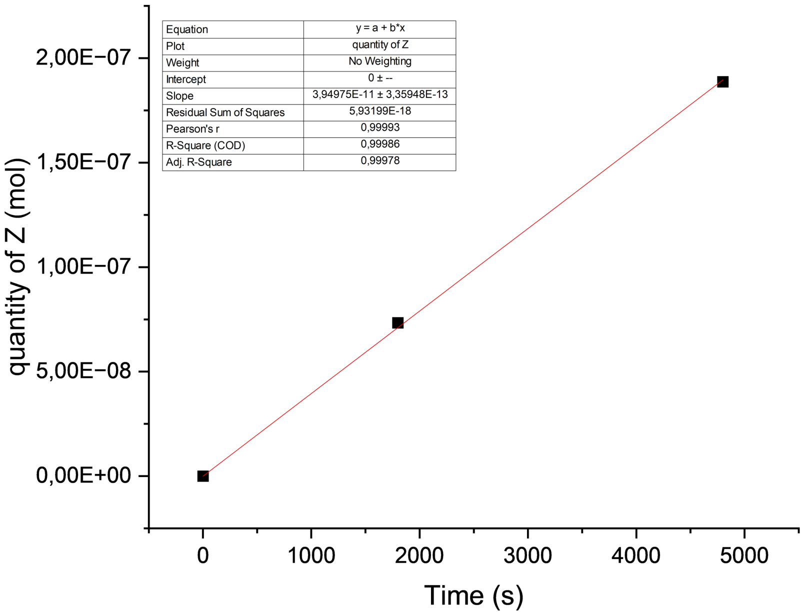

*Figure S21: Linear fit of the quantity of (Z)-isomer as a function of time obtained by irradiation of a **(E)-DTS-2** sample at 365 nm with a power of 3.97 mW.*

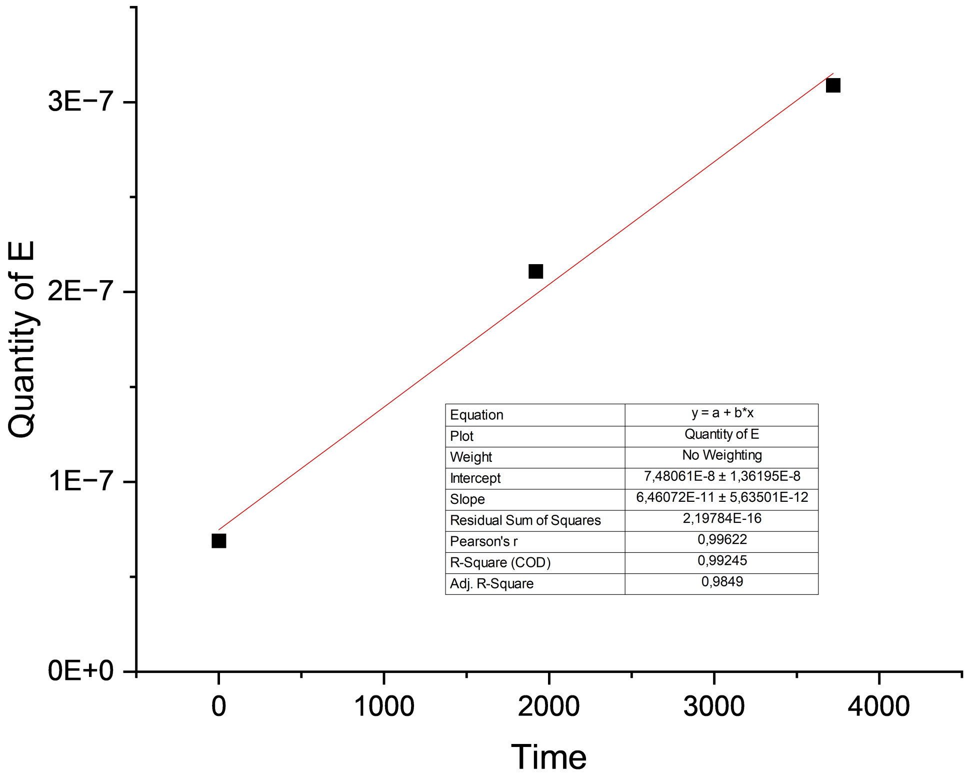

*Figure S22: Linear fit of the quantity of (Z)-isomer as a function of time obtained by irradiation of a **(Z)-DTS-2** sample at 365 nm with a power of 4.63 mW*

Luminescence quantum yield measurements

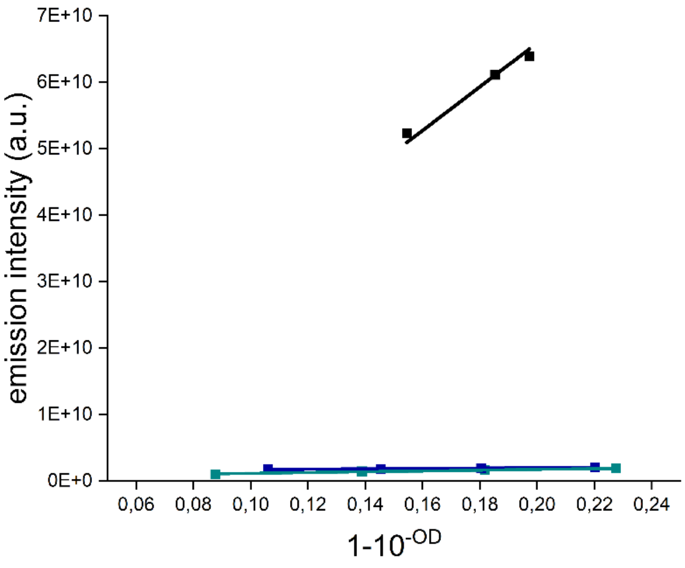

*Figure S23: Integrated emission area according to optical density of quinine sulphate in 0.5 M H_2SO_4 (black), **(E)-DTS-2** (blue) and **(Z)-DTS-2** (green) in THF at room temperature*

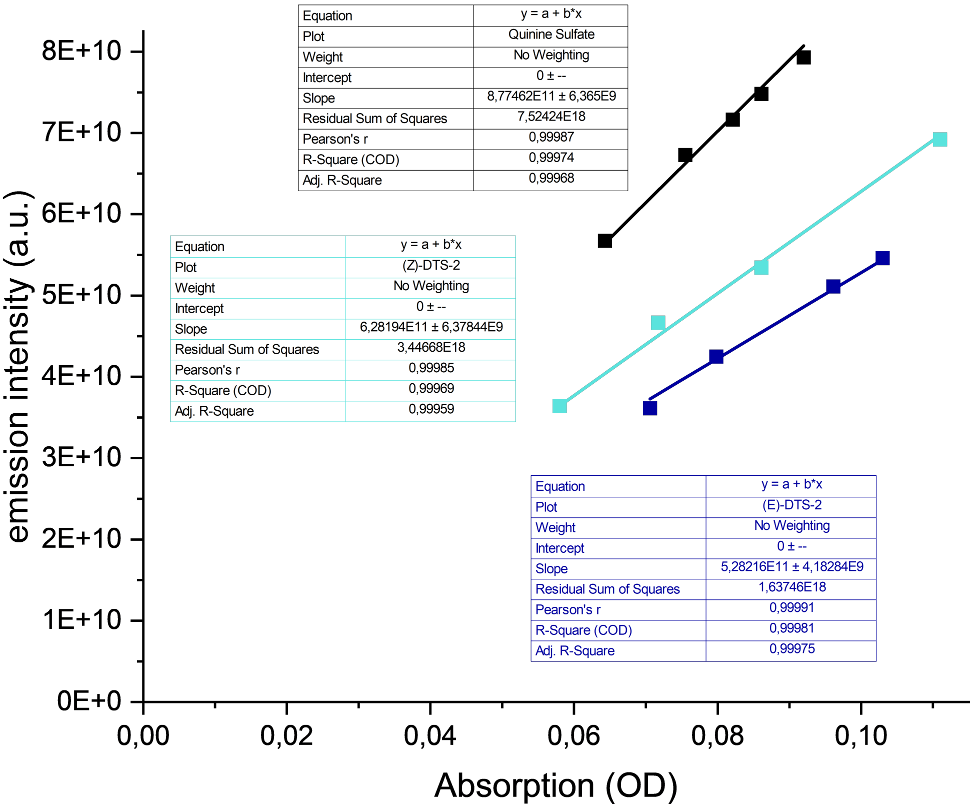

*Figure S24 . Integrated emission area according to optical density of quinine sulphate in 0.5 M H_2SO_4 (black), **(E)-DTS-2** (blue) and **(Z)-DTS-2** (cyan) in water/THF 90/10 mixture at room temperature*

Solvatochromism

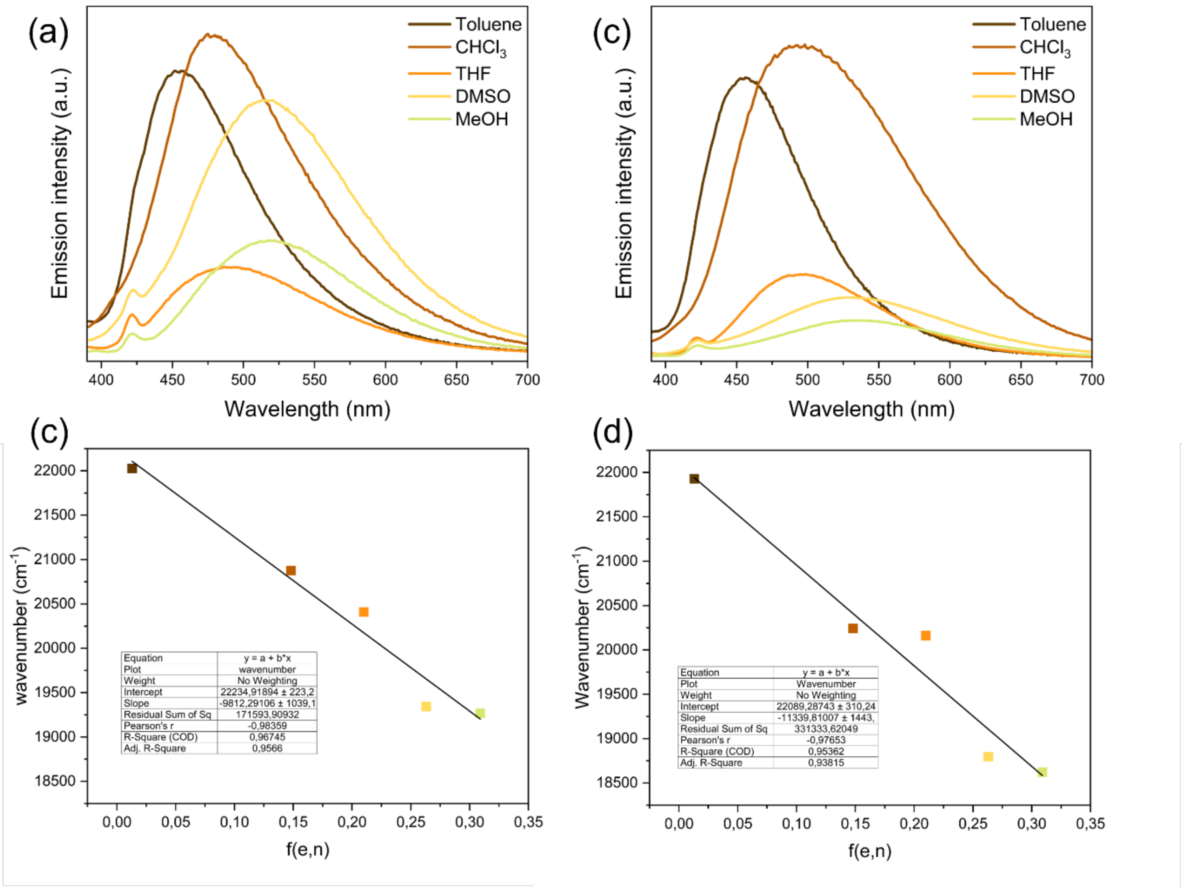

Figure S25: Emission spectra recorded in solvents of different polarity of ***(E)-DTS-2*** *(a) and* ***(Z)-DTS-2*** *(b). Wavenumber of the maxima of emission in different solvents as a fonction of the orientational polarizability (f(e,n)) of that solvent for* ***(E)-DTS-2*** *(c) and* ***(Z)-DTS-2*** *(d) and associated linear fit.*

Emission and absorption properties of DTS-Pyr and DTS-OH

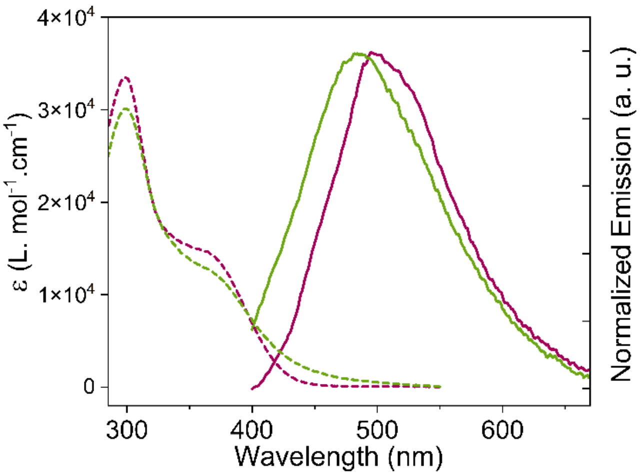

Figure S26: Absorption (dashed lines) and normalized emission (solid lines) for (E)-DTS-Pyr (pink curve), (E)-DTS-OH (green curve), in THF, 10^{-5} M.

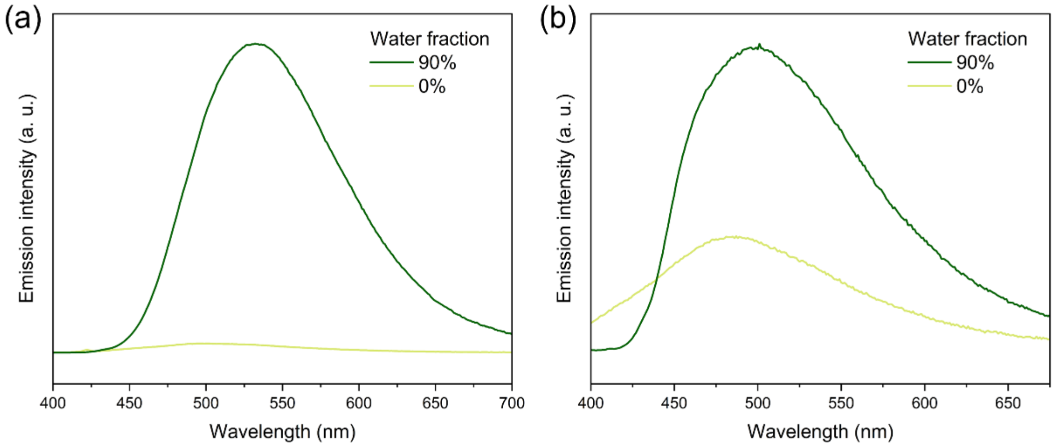

*Figure S27: Emission spectra of **(E)-DTS-Pyr** (a) and **(E)-DTS-OH** (b) with different THF/water fractions (f_w) at 10^{-5} M.*

Table S2: Experimental absorption and emission λ_{max} wavelengths in solution (THF) and in nanoprecipitates (90/10 water/THF) for the considered molecules.

compound	state	Absorption $\lambda_{max\ (nm)}$	Emission $\lambda_{max\ (nm)}$
(E)-DTS-Pyr	Solution	300, 367	501
	Nanoprecipitate	300, 374	532
(E)-DTS-OH	Solution	299, 357	484
	Nanoprecipitate	301, 372	498

XRD Powder diffraction

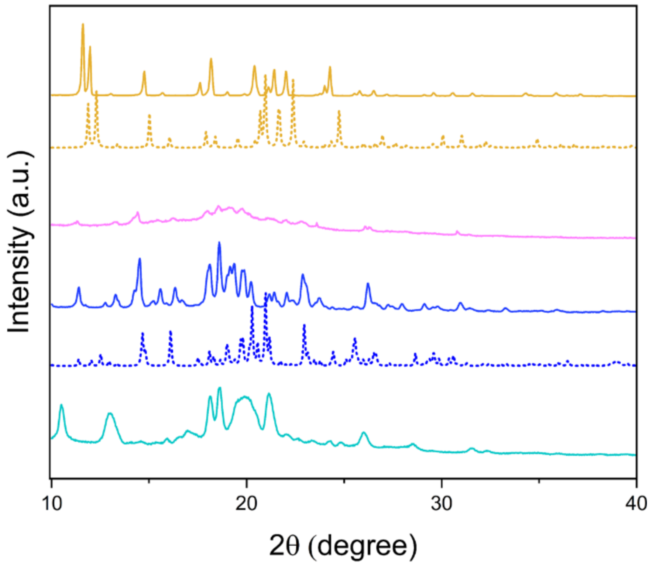

Figure S28: XRD Powder diffraction patterns for E-DTS-1 (yellow), E-DTS-2 in α (blue) and β (pink) and Z-DTS-2 (cyan); for crystalline samples, dotted lines feature the modelized theoretical XRD spectra calculated from single crystal data.

Emission in visqueous solvant

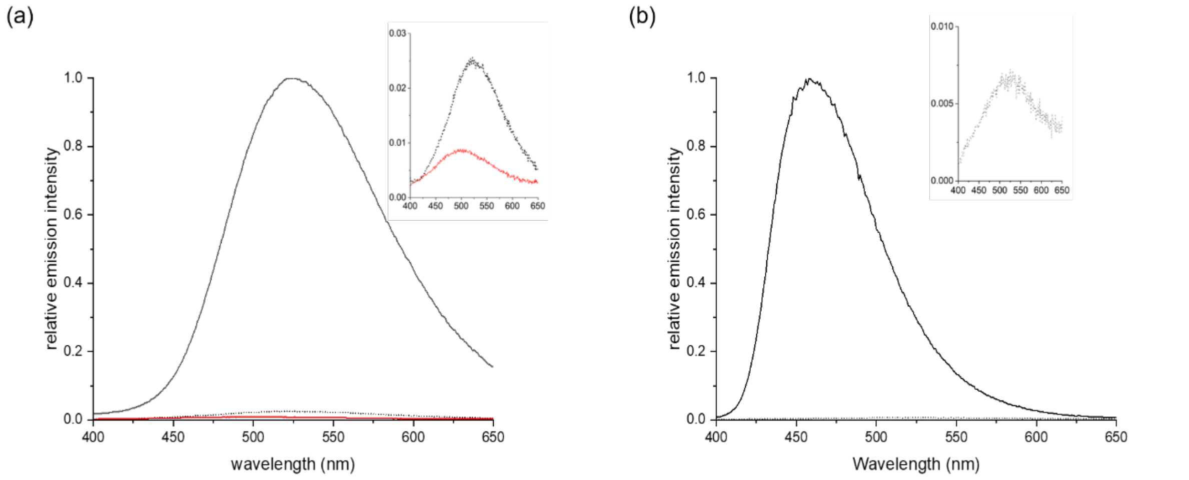

Figure 29: (a) Photoluminescence spectra of 10^{-5} M solutions of ***(E)-DTS-2*** *in THF (red), glycerol (black) and EtOH (black, dotted) ; inset : closeup of the data in THF and EtOH (b)) Photoluminescence spectra of 10^{-5} M solutions of* ***(E)-DTS-2*** *in Me-THF at 77K (black) and room temperature (black, dotted) ; inset : closeup of the data at room temperature.*

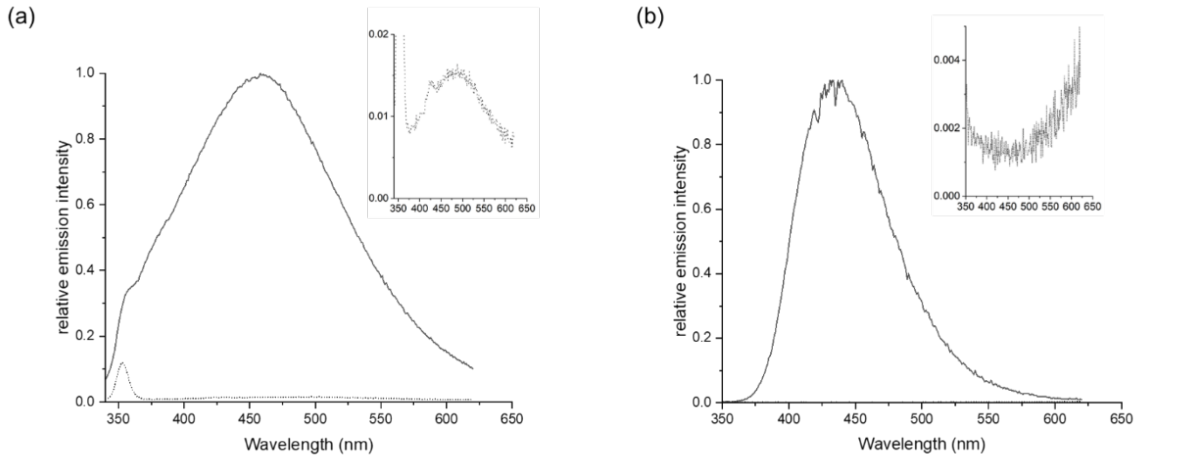

Figure S30: (a) Photoluminescence spectra of 10^{-5} M solutions of ***(E)-DTS-1*** *in glycerol (black) and EtOH (black, dotted) ; inset : closeup of the data in EtOH (b)) Photoluminescence spectra of 10^{-5} M solutions of* ***(E)-DTS-1*** *in Me-THF at 77K (black) and room temperature (black, dotted) ; inset : closeup of the data at room temperature.*

Computational methods

Molecular calculations were performed with ORCA 5.0.4 software[2]. To reduce calculation time, benzyl groups were substituted by methyl group as their pi electrons are not involved in any electronic transition for all molecules. All geometries were optimized using DFT or TD-DFT with the CAM-BL3YP[3] functional, the def2-SVP basis set, in PCM[4] THF as solvent and using the empirical dispersion correction D3-BJ[5]. Approximation of the resolution identity for Coulomb integrals and numerical integration for Hatree-Fock exchange with appropriate auxiliary basis were used as provided by the ORCA software. Absorption transitions were obtained using TDA-DFT with the CAM-B3LYP functional along with the PCM (THF) as solvent and with the def2-TZVP basis-set. Natural transition orbitals[6] were used to analyze those absorption transitions. Geometries at S_1 were obtained by a relaxed scan of the relevant dihedral angle from 0° to 180° per 10° steps using TD-DFT.

For crystalline structure, we performed geometry optimization at fixed cell parameters of both the ground state and the first excited state using periodic DFT and periodic TD-DFT with the CP2K software[7]. The functional used was PBE along with the MOLOPT DZVP-SR basis set and the GTH-PBE pseudo potentials[8]. The D3-BJ dispersion correction was used.

Natural transition orbitals

Energy of transition (eV)	f	hole	particule	NTO contribution
4.22	0.671	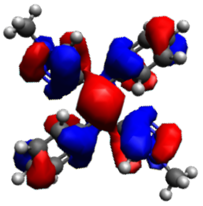	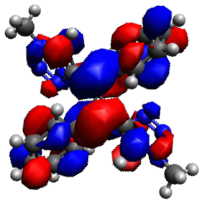	0.97

Table S3: NTO analysis for the main absorption transitions (f>0.1) of compound ***(E)-DTS-1*** *calculated at the camB3LYP/ def2-TZVP in CPCM(THF) with D3BJ dispersion.*

Energy of transition (eV)	f	hole	particule	NTO contribution
3.69	1.123	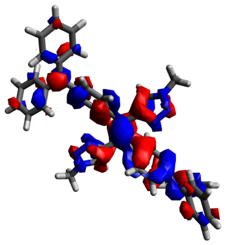	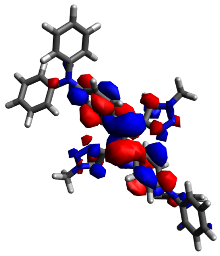	0.88
4.39	0.363	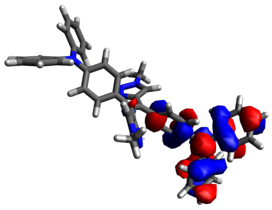	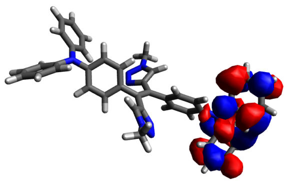	0.97
4.39	0.347	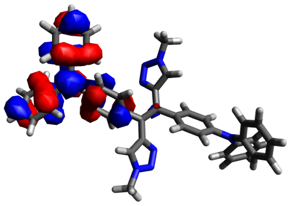	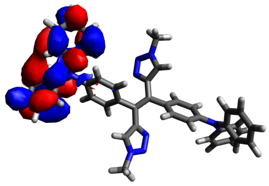	0.97
4.59	0.524	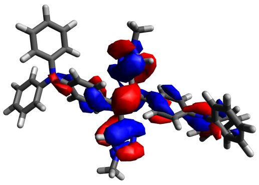	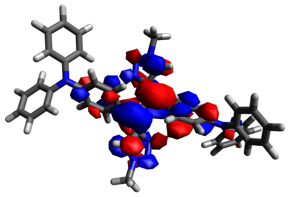	0.45

		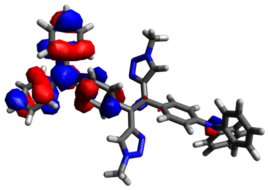	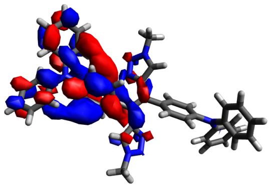	0.27
		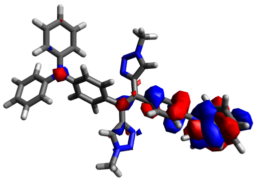	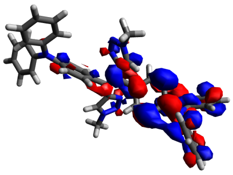	0.25

*Table S4: NTO analysis for the main absorption transitions (f>0.1) of compound **(E)-DTS-2** calculated at the camB3LYP/ def2-TZVP in CPCM(THF) with D3BJ dispersion.*

Energy of transition (eV)	f	hole	particule	NTO contribution
3.28	0.389	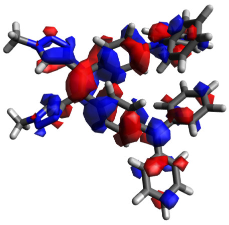	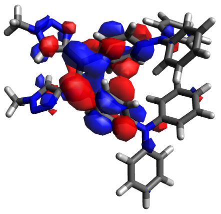	0.90
4.15	0.558	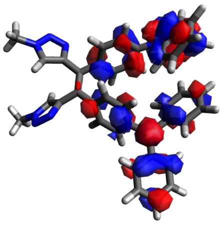	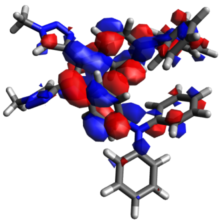	0.69
		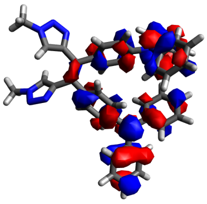	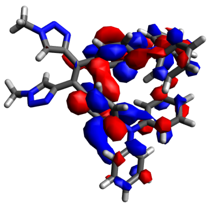	0.28

4.25	0.182	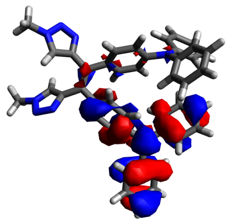	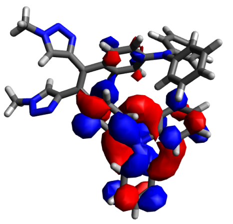	0.50
		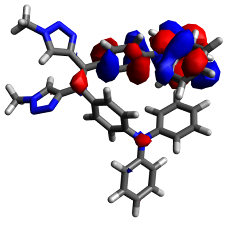	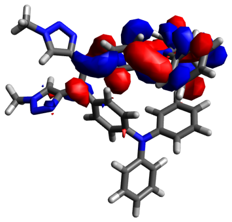	0.40
4.45	0.377	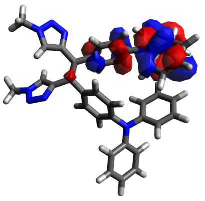	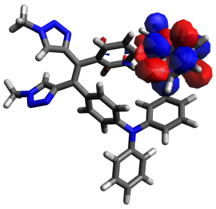	0.96
4.47	0.544	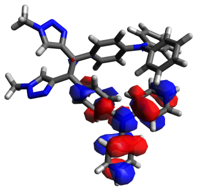	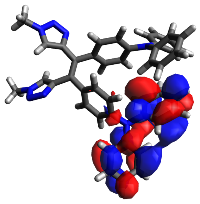	0.88

*Table S5: NTO analysis for the main absorption transitions (f>0.1) of compound **(Z)-DTS-2** calculated at the camB3LYP/ def2-TZVP in CPCM(THF) with D3BJ dispersion*

Computed absorption spectra.

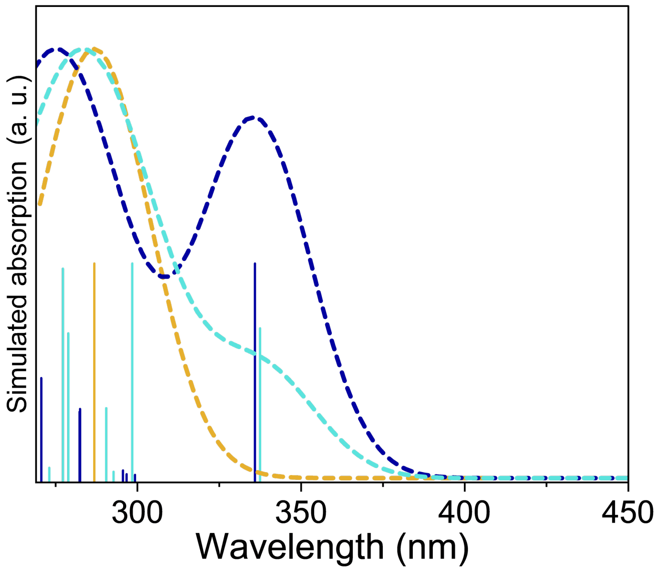

*Figure S31: TD-DFT simulated absorption spectra of **(E)-DTS-1** (yellow sticks), **(E)-DTS-2** (blue sticks), **(Z)-DTS-2** (cyan sticks) in CPCM(THF). TD-DFT computed transitions were convoluted with gaussian functions having a FWHM of 0.2 eV.*

Dihedral scans

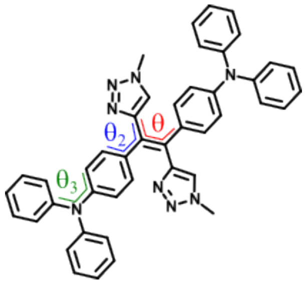

*Figure S32: Structure of molecule **DTS-2** as used in calculations. Dihedral angles used for scans are highlighted in color.*

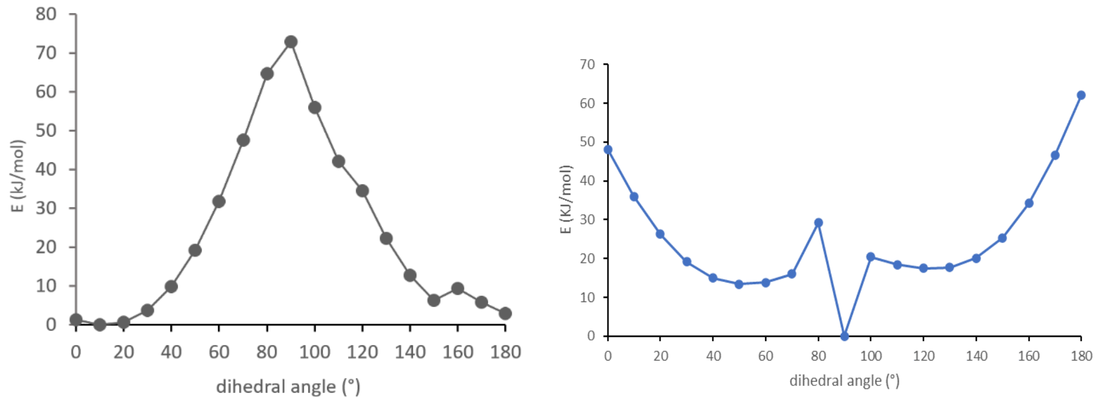

*Figure S33. Energy at the ground state (grey) and first excited state (blue) for different values of the dihedral angle θ of **DTS-1**.*

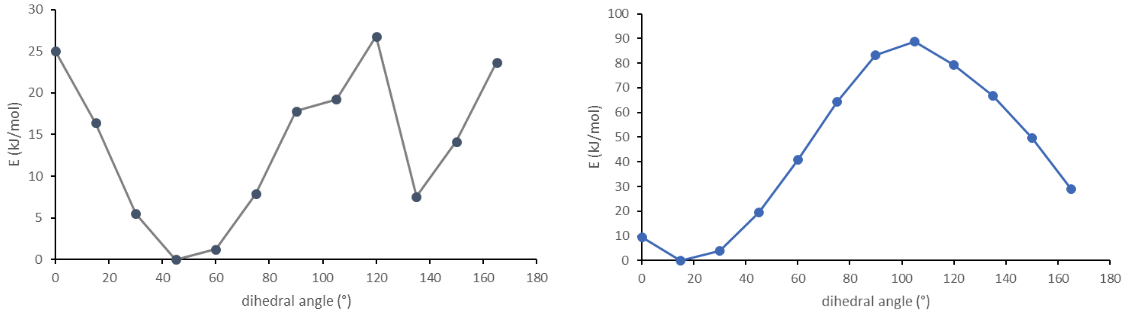

*Figure S34: Energy at the ground state (grey) and first excited state (blue) for different values of the dihedral angle θ_2 of **DTS-2**.*

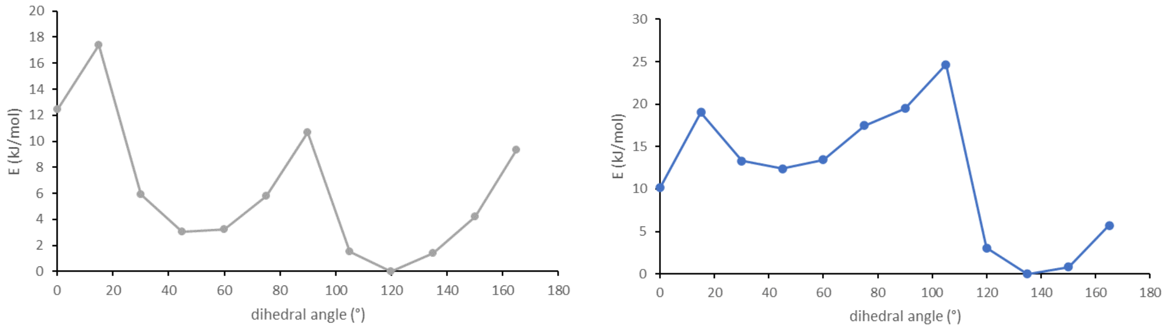

*Figure S35: Energy at the ground state (grey) and first excited state (blue) for different values of the dihedral angle θ_3 of **DTS-2**.*

	solution	crystal
**S_0**	169°	180°
**S_1**	124°	180°

*Table S6: Dihedral angle values (°) of the central carbon-carbon double bond **(E)-DTS-1** found at the minima of energy of the PES in solution (camB3LYP/def2-SVP) and in crystal*

Cartesian coordinates of computed structures

XYZ (E)-DTS-2 S_0

C	-0.39773426414935	0.77582550013898	0.10449945443068
C	0.37132868052174	-0.32500855818917	0.30252581927110
C	-1.87529395623977	0.67584251354575	-0.03213452836787
C	-2.73349490755791	1.39067256359255	0.80846224057634
H	-2.31541575313029	2.06751757077359	1.55677521076682
C	-4.10856127844046	1.20374282608166	0.75224192922430
H	-4.76073223591991	1.73229662113647	1.44903467530082
C	-4.66341995389274	0.31669892978440	-0.17664313695597
C	-3.81448403778626	-0.34136469948677	-1.07358537057073
H	-4.24001693333800	-1.00907202039728	-1.82411237911032
C	-2.44114712871255	-0.16962892635317	-0.99217121464204
H	-1.78796464742182	-0.71043721149596	-1.67964586744423
C	1.85322472371658	-0.31294900233489	0.18645249353732
C	2.66111469714702	-0.85273524194726	1.19277545288918
H	2.19690814236959	-1.28762280356611	2.08045969010702
C	4.04517362327490	-0.84200398078753	1.08788876926058
H	4.65443874477841	-1.26434750622375	1.88796532102901
C	4.66902684772337	-0.28396426209713	-0.03475969271843
C	3.86589771632464	0.23725067702400	-1.05694722009807
H	4.33248439047014	0.66711241879623	-1.94414689267728
C	2.48306487162816	0.21350595076502	-0.94666203389289
H	1.87522169844923	0.62952834829384	-1.75242421762805
N	6.07597863695631	-0.24708403357681	-0.12745200405362
N	-6.05503512129641	0.07022440001231	-0.20179624552719

C	-6.95845424811419	1.14666556614844	-0.05158944746150
C	-8.07561196303071	1.03565745209172	0.78485719956366
H	-8.25048671356624	0.10557299419469	1.32755312302712
C	-8.95632578342870	2.10348082136060	0.92480745201614
H	-9.82317605212133	1.99995127258580	1.58149946916945
C	-8.72990541683987	3.30208088222891	0.25040885957577
H	-9.41905351228858	4.14053844925572	0.36886538349678
C	-7.61285890179700	3.41853527513228	-0.57523559220817
H	-7.42398242591043	4.34993463332316	-1.11391179612753
C	-6.73829162617937	2.34895945126504	-0.73484343841214
H	-5.87224608052468	2.44084380027815	-1.39238702343228
C	-6.52412010636055	-1.25918483929524	-0.28934362507941
C	-7.68440352313441	-1.55733230719043	-1.01572893682752
H	-8.22462465978734	-0.75420885888362	-1.51885031823534
C	-8.14711497562868	-2.86654875983456	-1.09716109414692
H	-9.05452132613017	-3.07822090820026	-1.66744701051887
C	-7.45502520744600	-3.90404381513892	-0.47477050438685
H	-7.81597559109363	-4.93178103744128	-0.54809578199112
C	-6.29460826618584	-3.61209774494812	0.24006653308686
H	-5.74031128117722	-4.41184340573451	0.73669083152132
C	-5.83432688277338	-2.30354333881199	0.34229584705483
H	-4.92630348225364	-2.08985711367148	0.90789292764978
C	6.84848076364592	0.05338780896443	1.02021080076222
C	8.01104755609665	-0.67368286636888	1.30122467725474
H	8.31402136187249	-1.48062674075328	0.63222212666519
C	8.77487917196572	-0.36963566661766	2.42356736757877
H	9.68022693524611	-0.94567742175484	2.62807060347016
C	8.38296640008143	0.64824061048095	3.29133543974040
H	8.98046677187257	0.87969773866491	4.17532575517032
C	7.22076835246715	1.36783675050527	3.01815498130436
H	6.90544810537988	2.17280098642779	3.68581642592646
C	6.46168919802519	1.08136900235567	1.88808869419826
H	5.55892256799814	1.65528966189798	1.67278389681810
C	6.71929688991273	-0.46955644486629	-1.36611830426569
C	6.28733667608452	-1.49129000220709	-2.22093453491808
H	5.44614813520713	-2.12012318646955	-1.92467130643072
C	6.92543940808252	-1.70446066448104	-3.43858681358627
H	6.57693026731685	-2.50661174868978	-4.09311610555773
C	8.01137119270661	-0.91663802104047	-3.81741892027704
H	8.51505826548071	-1.09131788330090	-4.77020338493034
C	8.44712942786291	0.09668655543713	-2.96561273905558
H	9.29232659880303	0.72699613404455	-3.25133417429898
C	7.80303507015013	0.32744304368728	-1.75450647407479
H	8.13991524456120	1.13154095825504	-1.09873902277054
C	-0.22201938792352	-1.63300360208881	0.63750709203331
C	0.19161050339896	-2.89292747615373	0.24457346355375
N	-0.68430622030212	-3.73585729695765	0.82770761844104
H	1.01082204515838	-3.23367267013006	-0.38224274876714

N	-1.30724572448145	-1.79996637505478	1.44541868833810
N	-1.57743519783986	-3.05887767838746	1.54804762900443
C	0.15637230901280	2.13578632443604	0.01313656573787
C	-0.31492333071146	3.20112066575381	-0.73308681602370
N	0.52747861655559	4.21407164669203	-0.44857762538193
H	-1.15180656741241	3.30344139455274	-1.41802346591663
N	1.24018951910665	2.58058381185196	0.71026483866309
N	1.45441432307750	3.82140165615567	0.42438907189938
C	-0.74651886361140	-5.18039124764192	0.74356866750299
H	0.22440558947093	-5.61462409708598	1.01453565977741
H	-1.01901198731131	-5.49400063130367	-0.27316953940274
H	-1.51211845515423	-5.51840879021375	1.45030313890343
C	0.51981724154071	5.56925155435975	-0.95964181029326
H	-0.43691894738186	6.05589390716884	-0.72922994689741
H	0.67841987806282	5.56772099282073	-2.04623767549893
H	1.33631576622293	6.11001076485197	-0.46913510443677

XYZ (Z)-DTS-2 S_0

C	-3.005791	-1.141753	0.057176
C	-3.179176	0.200851	-0.049917
C	-1.638860	-1.719938	0.190215
C	-1.150108	-2.643143	-0.739036
H	-1.811655	-3.026906	-1.518787
C	0.180103	-3.041588	-0.713283
H	0.560165	-3.735242	-1.465123
C	1.055330	-2.531520	0.251456
C	0.554092	-1.668491	1.230324
H	1.221140	-1.279498	2.001106
C	-0.776371	-1.275395	1.197788
H	-1.143652	-0.566580	1.941646
C	-2.019711	1.113509	-0.239796
C	-1.826792	2.220158	0.594104
H	-2.577185	2.463095	1.349523
C	-0.658748	2.971310	0.525492
H	-0.495043	3.798305	1.218383
C	0.333087	2.644299	-0.402842
C	0.104454	1.599654	-1.302658
H	0.860537	1.367483	-2.054645
C	-1.055487	0.845911	-1.218982
H	-1.207700	0.016091	-1.909372
N	1.585182	3.310029	-0.412719
N	2.432955	-2.858714	0.199298
C	3.125053	-2.629103	-1.016210
C	4.056648	-3.558030	-1.491939
H	4.242887	-4.469997	-0.922337
C	4.743830	-3.316578	-2.677358
H	5.471656	-4.048713	-3.034390

C	4.497639	-2.158811	-3.414242
H	5.035150	-1.974595	-4.346604
C	3.558961	-1.239563	-2.949184
H	3.362061	-0.323679	-3.511118
C	2.881160	-1.466985	-1.756279
H	2.165018	-0.734526	-1.383485
C	3.125805	-3.219443	1.370141
C	4.462321	-2.842592	1.567970
H	4.978521	-2.262520	0.803182
C	5.129969	-3.198787	2.735190
H	6.169453	-2.889985	2.868433
C	4.482891	-3.925113	3.733077
H	5.008663	-4.197378	4.650219
C	3.154237	-4.300288	3.540831
H	2.631750	-4.876551	4.307906
C	2.482201	-3.961459	2.371355
H	1.446150	-4.271387	2.228644
C	1.649014	4.713703	-0.423233
C	2.674879	5.401738	0.241406
H	3.439714	4.843358	0.781811
C	2.719559	6.792151	0.218223
H	3.527947	7.307130	0.742686
C	1.741345	7.526671	-0.448950
H	1.777445	8.617691	-0.458469
C	0.715263	6.846440	-1.103179
H	-0.059068	7.403962	-1.635389
C	0.669748	5.456877	-1.100306
H	-0.131659	4.936660	-1.626616
C	2.740457	2.512843	-0.212354
C	2.731519	1.500200	0.753723
H	1.834508	1.339656	1.353050
C	3.852448	0.699402	0.944795
H	3.826371	-0.090635	1.697803
C	5.001176	0.902736	0.183489
H	5.879598	0.271462	0.332601
C	5.014996	1.912675	-0.777856
H	5.906443	2.075312	-1.387730
C	3.892114	2.709183	-0.982702
H	3.900877	3.491022	-1.744142
C	-4.502026	0.844256	0.048926
C	-5.482978	0.701882	1.011340
N	-6.456036	1.551726	0.628103
H	-5.555676	0.081507	1.900216
N	-4.937448	1.777184	-0.841100
N	-6.112283	2.189385	-0.488998
C	-4.118604	-2.103939	0.012633
C	-5.299808	-2.077813	-0.708292
N	-5.906766	-3.240238	-0.403174

H	-5.731575	-1.350954	-1.389999
N	-4.087616	-3.283821	0.692087
N	-5.164149	-3.953934	0.441599
C	-7.736632	1.802602	1.257252
H	-7.593523	2.018662	2.323549
H	-8.396447	0.931798	1.143941
H	-8.183948	2.670529	0.760893
C	-7.187268	-3.736784	-0.863998
H	-7.221467	-3.724974	-1.960913
H	-8.002477	-3.118645	-0.464577
H	-7.292168	-4.765059	-0.501723

XYZ (E)-DTS-2 S_1

C	-0.389914	-0.525039	1.220591
C	0.461734	-1.276451	0.294161
C	-1.715489	-0.155059	0.843628
C	-2.714456	0.276748	1.765110
H	-2.455282	0.364036	2.817484
C	-4.000923	0.560515	1.363494
H	-4.741015	0.856299	2.108284
C	-4.386254	0.451968	0.010484
C	-3.409159	0.059723	-0.922736
H	-3.670554	-0.002789	-1.979826
C	-2.125758	-0.239286	-0.521230
H	-1.388925	-0.513286	-1.276700
C	1.764801	-0.794089	-0.036462
C	2.773397	-1.582454	-0.664363
H	2.536020	-2.602002	-0.958191
C	4.031068	-1.083110	-0.922152
H	4.772520	-1.722864	-1.402736
C	4.378565	0.241339	-0.583173
C	3.392826	1.046400	0.017696
H	3.627818	2.077562	0.284091
C	2.136283	0.546609	0.282912
H	1.396531	1.203105	0.741811
N	5.661331	0.735358	-0.836698
N	-5.698885	0.711574	-0.392009
C	-6.472605	1.703728	0.257099
C	-7.808546	1.450638	0.589664
H	-8.245887	0.478989	0.355266
C	-8.571148	2.431376	1.215050
H	-9.612440	2.220808	1.468800
C	-8.011239	3.668629	1.530044
H	-8.610903	4.434532	2.025797
C	-6.678936	3.919588	1.204746
H	-6.230816	4.887725	1.439071
C	-5.914057	2.949743	0.565496

H	-4.876085	3.154080	0.298513
C	-6.278669	-0.029833	-1.451245
C	-7.007593	0.621944	-2.451987
H	-7.116944	1.707005	-2.416048
C	-7.586275	-0.109826	-3.483904
H	-8.152533	0.410847	-4.259311
C	-7.434411	-1.494398	-3.539883
H	-7.884639	-2.065347	-4.354310
C	-6.703654	-2.144298	-2.545818
H	-6.584569	-3.229699	-2.574411
C	-6.134810	-1.421488	-1.502986
H	-5.574300	-1.932594	-0.718566
C	6.791996	-0.111234	-0.731223
C	7.800282	-0.064370	-1.700654
H	7.701568	0.619213	-2.545374
C	8.918427	-0.883505	-1.586254
H	9.698293	-0.836772	-2.349612
C	9.040021	-1.768568	-0.516119
H	9.916285	-2.414401	-0.432226
C	8.033024	-1.820892	0.446609
H	8.120217	-2.504403	1.294151
C	6.919427	-0.993862	0.347771
H	6.139890	-1.025156	1.110575
C	5.854082	2.099122	-1.168425
C	5.027287	2.722353	-2.110311
H	4.234154	2.147850	-2.591346
C	5.218546	4.062217	-2.429735
H	4.566586	4.536672	-3.166498
C	6.243263	4.792755	-1.829222
H	6.394636	5.842883	-2.086747
C	7.073225	4.170359	-0.898197
H	7.876233	4.733421	-0.417432
C	6.878290	2.834690	-0.561766
H	7.521230	2.352429	0.176105
C	-0.083647	-2.521964	-0.191711
C	-1.246438	-3.162930	0.247297
N	-1.355275	-4.252481	-0.532887
H	-1.959297	-2.926409	1.030503
N	0.412308	-3.289239	-1.218226
N	-0.351303	-4.311210	-1.408539
C	0.176056	-0.261964	2.523364
C	1.346845	-0.803541	3.061087
N	1.468535	-0.228816	4.270722
H	2.057291	-1.530449	2.680711
N	-0.310684	0.612092	3.465216
N	0.465094	0.619871	4.495690
C	-2.394243	-5.260042	-0.534149
H	-3.353732	-4.816187	-0.833375

H	-2.491616	-5.702855	0.465647
H	-2.104474	-6.032626	-1.254471
C	2.515197	-0.409123	5.254227
H	3.474229	-0.046784	4.859696
H	2.605738	-1.470794	5.518803
H	2.238210	0.170639	6.141316

XYZ (Z)-DTS-2 S_1

C	-2.918273	-1.090739	-0.174193
C	-3.123962	0.305733	0.189997
C	-1.651581	-1.703539	0.087400
C	-1.142094	-2.814425	-0.647591
H	-1.786984	-3.313764	-1.367892
C	0.157400	-3.245247	-0.496018
H	0.525739	-4.072012	-1.105691
C	1.032100	-2.631004	0.426166
C	0.527114	-1.589891	1.223230
H	1.171686	-1.114426	1.963834
C	-0.763266	-1.138171	1.052087
H	-1.117346	-0.316780	1.674161
C	-2.102251	1.257818	-0.113118
C	-1.924105	2.488110	0.586593
H	-2.672128	2.793925	1.315646
C	-0.788970	3.252529	0.427421
H	-0.653440	4.146251	1.038221
C	0.230384	2.863978	-0.469932
C	0.012471	1.726279	-1.265790
H	0.753775	1.446817	-2.015678
C	-1.106464	0.947938	-1.088671
H	-1.245431	0.073805	-1.723374
N	1.452356	3.538579	-0.539993
N	2.369766	-3.036354	0.513489
C	3.090684	-3.292028	-0.681142
C	3.901899	-4.425993	-0.790276
H	3.965729	-5.122637	0.047378
C	4.621477	-4.657681	-1.957823
H	5.252651	-5.545877	-2.033097
C	4.529356	-3.772483	-3.031424
H	5.091933	-3.960123	-3.948135
C	3.714884	-2.646031	-2.925409
H	3.642126	-1.940937	-3.756391
C	3.002808	-2.400687	-1.756200
H	2.380052	-1.509570	-1.664243
C	3.044299	-3.056152	1.753309
C	4.379825	-2.642173	1.848495
H	4.905243	-2.302665	0.955259
C	5.035579	-2.666037	3.075124

H	6.075980	-2.338057	3.131220
C	4.373321	-3.089698	4.225732
H	4.889526	-3.101384	5.187561
C	3.044246	-3.501721	4.133191
H	2.514518	-3.846848	5.023945
C	2.384716	-3.494555	2.909564
H	1.349711	-3.832223	2.841647
C	1.529387	4.939422	-0.385727
C	2.578577	5.519105	0.339116
H	3.336564	4.880245	0.794178
C	2.650603	6.900593	0.481030
H	3.474483	7.337781	1.049539
C	1.675474	7.723182	-0.081135
H	1.732237	8.806888	0.037984
C	0.627640	7.147123	-0.798026
H	-0.139200	7.779445	-1.251064
C	0.555757	5.767918	-0.958828
H	-0.256912	5.323356	-1.535137
C	2.636459	2.773665	-0.704239
C	2.828967	1.612430	0.054993
H	2.074392	1.315927	0.784929
C	3.966423	0.835890	-0.129972
H	4.098824	-0.070538	0.463722
C	4.935131	1.215153	-1.059069
H	5.827919	0.603481	-1.203310
C	4.753903	2.381238	-1.801284
H	5.504908	2.686438	-2.533276
C	3.609498	3.155449	-1.633020
H	3.459804	4.057739	-2.228359
C	-4.336368	0.656963	0.893675
C	-5.225220	-0.192558	1.553119
N	-6.197306	0.617434	2.012560
H	-5.219870	-1.264623	1.724769
N	-4.855072	1.923202	1.004341
N	-5.958559	1.884081	1.672730
C	-3.987742	-1.776781	-0.861692
C	-5.098444	-1.220204	-1.497821
N	-5.795638	-2.275826	-1.957881
H	-5.411673	-0.192542	-1.654619
N	-4.112472	-3.138639	-0.985442
N	-5.188745	-3.419279	-1.640145
C	-7.391113	0.265397	2.751472
H	-7.122918	-0.303805	3.651065
H	-8.064468	-0.336129	2.125522
H	-7.889185	1.197720	3.038744
C	-7.050743	-2.283468	-2.678728
H	-6.974979	-1.651787	-3.573390
H	-7.862167	-1.913252	-2.037207

H	-7.256628	-3.318235	-2.973133

XYZ DTS-2 at θ=90° S_1

C	-0.46909	-1.16305	1.70987
C	0.55588	-1.83988	0.84127
C	-1.60224	-0.61045	1.08119
C	-2.68559	0.01903	1.78671
H	-2.63009	0.06742	2.87124
C	-3.78817	0.54113	1.13076
H	-4.58660	1.00377	1.71978
C	-3.91908	0.50050	-0.26215
C	-2.88018	-0.09691	-0.98153
H	-2.95235	-0.14473	-2.07250
C	-1.77315	-0.63882	-0.34915
H	-1.00373	-1.09573	-0.97799
C	1.66164	-1.16012	0.37342
C	2.70733	-1.73141	-0.44588
H	2.62734	-2.77467	-0.74706
C	3.76947	-0.99712	-0.84470
H	4.54075	-1.44880	-1.46454
C	3.87053	0.39898	-0.48693
C	2.83232	0.98866	0.31120
H	2.90491	2.03335	0.60493
C	1.78957	0.23123	0.73137
H	1.00391	0.64600	1.36334
N	4.91442	1.12144	-0.88782
N	-5.07756	1.02857	-0.92490
C	-5.27815	2.41749	-0.93349
C	-6.56578	2.97998	-0.90212
H	-7.43889	2.32728	-0.88130
C	-6.73863	4.36002	-0.88813
H	-7.75186	4.76867	-0.86169
C	-5.64002	5.21817	-0.89019
H	-5.78020	6.30085	-0.87430
C	-4.35932	4.66728	-0.90454
H	-3.48319	5.32062	-0.90272
C	-4.17629	3.28906	-0.92697
H	-3.17068	2.86860	-0.93421
C	-5.98175	0.13730	-1.52303
C	-6.70841	0.48488	-2.67516
H	-6.58061	1.47448	-3.11483
C	-7.58275	-0.42146	-3.26556
H	-8.13293	-0.12295	-4.16145
C	-7.74960	-1.70121	-2.73858
H	-8.43437	-2.41088	-3.20715
C	-7.01962	-2.05919	-1.60606
H	-7.13153	-3.05816	-1.17702

C	-6.14860	-1.15788	-1.00406
H	-5.57990	-1.45069	-0.12252
C	6.06921	0.52542	-1.50965
C	6.34914	0.81137	-2.84358
H	5.68176	1.46297	-3.41007
C	7.47818	0.25615	-3.43710
H	7.69947	0.47290	-4.48375
C	8.32051	-0.57420	-2.69887
H	9.20591	-1.00838	-3.16728
C	8.03491	-0.84861	-1.36296
H	8.69465	-1.49519	-0.78140
C	6.90857	-0.29550	-0.76089
H	6.67591	-0.50035	0.28602
C	4.96238	2.55227	-0.72237
C	4.10673	3.35736	-1.47047
H	3.39440	2.89810	-2.15871
C	4.17089	4.73977	-1.32475
H	3.50050	5.37529	-1.90623
C	5.08923	5.30781	-0.44376
H	5.13824	6.39276	-0.33233
C	5.94661	4.49295	0.29399
H	6.66622	4.93627	0.98473
C	5.88794	3.10968	0.15569
H	6.55298	2.46054	0.72778
C	0.28006	-3.22866	0.57235
C	-0.84995	-3.89505	1.04570
N	-0.74161	-5.12883	0.56621
H	-1.67113	-3.55108	1.66692
N	0.99045	-4.13364	-0.17874
N	0.38834	-5.25431	-0.17613
C	-0.15580	-1.26196	3.10470
C	0.99614	-1.80498	3.69595
N	0.80287	-1.66532	5.03930
H	1.89618	-2.25426	3.28804
N	-0.95072	-0.85232	4.15581
N	-0.35140	-1.09918	5.29355
C	-1.63631	-6.26039	0.72116
H	-2.11117	-6.48920	-0.24156
H	-2.40117	-6.00125	1.46114
H	-1.06151	-7.12814	1.06634
C	1.68751	-2.05522	6.11060
H	2.64695	-1.52410	6.03272
H	1.87458	-3.13802	6.08169
H	1.20007	-1.79322	7.05640

References

1 S. Hwang, H. Bae, S. Kim and S. Kim, *Tetrahedron*, 2012, **68**, 1460–1465.
2 F. Neese, *WIREs Comput. Mol. Sci.*, 2022, **12**, e1606.
3 T. Yanai, D. P. Tew and N. C. Handy, *Chem. Phys. Lett.*, 2004, **393**, 51–57.
4 M. Garcia-Ratés and F. Neese, *J. Comput. Chem.*, 2020, **41**, 922–939.
5 S. Grimme, S. Ehrlich and L. Goerigk, *J. Comput. Chem.*, 2011, **32**, 1456–1465.
6 R. L. Martin, *J. Chem. Phys.*, 2003, **118**, 4775–4777.
7 T. D. Kühne, M. Iannuzzi, M. Del Ben, V. V. Rybkin, P. Seewald, F. Stein, T. Laino, R. Z. Khaliullin, O. Schütt, F. Schiffmann, D. Golze, J. Wilhelm, S. Chulkov, M. H. Bani-Hashemian, V. Weber, U. Borštnik, M. Taillefumier, A. S. Jakobovits, A. Lazzaro, H. Pabst, T. Müller, R. Schade, M. Guidon, S. Andermatt, N. Holmberg, G. K. Schenter, A. Hehn, A. Bussy, F. Belleflamme, G. Tabacchi, A. Glöß, M. Lass, I. Bethune, C. J. Mundy, C. Plessl, M. Watkins, J. VandeVondele, M. Krack and J. Hutter, *J. Chem. Phys.*, 2020, **152**, 194103.
8 W.-L. Li, K. Chen, E. Rossomme, M. Head-Gordon and T. Head-Gordon, *J. Phys. Chem. Lett.*, 2021, **12**, 10304–10309.
